# HIF-2α/LINC02609/APOL1-mediated lipid storage promotes endoplasmic reticulum homeostasis and regulates tumor progression in clear-cell renal cell carcinoma

**DOI:** 10.1186/s13046-023-02940-6

**Published:** 2024-01-23

**Authors:** Haibing Xiao, Yan Qu, Haolin Li, Yi Zhang, Mintian Fei, Chaozhao Liang, Hongmei Yang, Xiaoping Zhang

**Affiliations:** 1 Department of Urology, Institute of Urology, Anhui Province Key Laboratory of Genitourinary Diseases, The First Affiliated Hospital of Anhui Medical University, Anhui Medical University, Hefei, 230022 China; 2 Department of Urology, Tongji Medical College, Union Hospital, Huazhong University of Science and Technology, 1277 Jiefang Avenue, Wuhan, Hubei Province 430022 China; 3 Department of Pathogenic Biology, School of Basic Medicine, Tongji Medical College, Huazhong University of Science and Technology, Wuhan, 430030 China; 4 College of Basic Medicine, Xinxiang Medical University, Xinxiang, Henan 453000 China

**Keywords:** ccRCC, HIF-2α, APOL1, LINC02609, miR-149-5p, Lipid storage

## Abstract

**Background:**

The VHL-HIF pathway and lipid droplet accumulation are the main characteristics of clear cell renal cell carcinoma (ccRCC). However, the connection between the two features is largely unknown.

**Methods:**

We used transcriptional sequencing and TCGA database analysis to identify APOL1 as a novel therapeutic target for ccRCC. The oncogenic functions of APOL1 were investigated by cell proliferation, colony formation, migration and invasion assays in ccRCC cells in vitro and xenografts derived from ccRCC cells in vivo. Oil red O staining and quantification were used to detect lipid droplets. Chromatin immunoprecipitation (ChIP) assays and luciferase reporter assays were carried out to identify HIF-2α bound to the promoter of APOL1 and lncRNA LINC02609. RNA-FISH and luciferase reporter assays were performed to determine that LncRNA LINC02609 functions as a competing endogenous RNA to regulate APOL1 expression by sponging miR-149-5p.

**Findings:**

RNA-seq data revealed that HIF2α can regulate APOL1 and lncRNA LINC02609 expression. We also found that HIF-2α can bind to the promoter of APOL1 and lncRNA LINC02609 and transcriptionally regulate their expression directly. We further demonstrated that LncRNA LINC02609 functions as a competing endogenous RNA to regulate APOL1 expression by sponging miR-149-5p in ccRCC. Mechanistically, APOL1-dependent lipid storage is required for endoplasmic reticulum (ER) homeostasis and cell viability and metastasis in ccRCC. We also showed that high APOL1 expression correlated with worse clinical outcomes, and knockdown of APOL1 inhibited tumor cell lipid droplet formation, proliferation, metastasis and xenograft tumor formation abilities. Together, our studies identify that HIF2α can regulate the expression of the lipid metabolism related gene APOL1 by direct and indirect means, which are essential for ccRCC tumorigenesis.

**Interpretation:**

Based on the experimental data, in ccRCC, the HIF-2α/LINC02609/APOL1 axis can regulate the expression of APOL1, thus interfering with lipid storage, promoting endoplasmic reticulum homeostasis and regulating tumor progression in ccRCC. Together, our findings provide potential biomarkers and novel therapeutic targets for future studies in ccRCC.

**Supplementary Information:**

The online version contains supplementary material available at 10.1186/s13046-023-02940-6.

## Introduction

Renal cell carcinoma (RCC) accounts for nearly 5% of adult malignancies with about 431,288 new cases and 179,368 deaths estimated in 2020 with a focus on geographic variability across 20 world regions [1]. Clear cell renal cell carcinoma (ccRCC) is the most common subtype of RCC and accounts for approximately 75–80% of these tumors [2]. The most typical character of ccRCC is the presence of intracellular lipid droplets (LDs), which consist of a neutral lipid core containing triglycerides and cholesterol-esters surrounded by a phospholipid monolayer and associated LD surface proteins. This is also why it is named clear cell renal cell carcinoma (ccRCC) [3]. As such, reprogramming lipid metabolism may play pivotal roles in providing energy, macromolecules for membrane synthesis, and lipid-mediated signaling during cancer progression [4]. Why is there a large number of lipid droplets? Are droplets waste generated by the rapid growth of tumors or do they play an important role in the occurrence and development of tumors?

Another important character of ccRCC must be the very high frequency of biallelic VHL inactivation caused by allelic deletion or loss of heterozygosity on chromosome 3p (> 90%) along with gene mutation (~ 50%) or promoter hypermethylation (5–10%) [5]. A very elegant series of studies have shown that the VHL complex targets the hypoxia-inducible factors, HIF1α and HIF2α, for ubiquitin-mediated degradation in an oxygen-sensitive fashion [6, 7]. HIF is composed of an α subunit (HIF-1α, HIF-2α, and HIF-3α subunits) and a β subunit (HIF-1β/ARNT). Whereas HIF-1β is constitutively present, the HIF-α member is highly unstable, except under low oxygen concentrations. Both HIF-1α and HIF-2α can activate transcription [8]. Hypoxia-inducible Factor 1α (HIF1α) and HIF2α are broadly expressed in many human cancers, and the expression of these proteins frequently correlates with poor patient prognosis. Surprisingly, HIF1α have a low expression and function as a tumor suppressor in renal cell carcinoma [8–11]. Additionally, small molecule inhibitors targeting HIF2α have been developed with promising results in some patients with ccRCC. Similar to PT2399, a selective HIF-2 antagonist had greater activity than sunitinib, was active in sunitinib-progressing tumors, and was better tolerated in patients [12, 13].

Apolipoprotein L1 (APOL1) is a protein encoded by the APOL1 gene. It consists of two variants APOL1G1 and APOL1G2 [14]. The APOL1G1 variant comprises two substitution mutations at amino acid position 342 (S→G) and at amino acid position 384 (I→M), while the APOL1G2 variant comprises two amino acids deletion (388 N 389 Y) [15]. Both variants increase the risk for chronic kidney disease (CKD) and end-stage renal disease (ESRD) in patients of Sub-Saharan African descent [16]. The expression of APOL1 risk alleles is causal for altered podocyte function and glomerular disease [17]. The intracellular localization and function of APOL1 in podocytes are still unclear, and recent studies have suggested that APOL1 may play important role in the endoplasmic reticulum (ER), mitochondria, endosomes, lysosomes, and autophagosomes [18, 19], Justin Chun et al. even demonstrated that APOL1 also localizes to intracellular lipid droplets (LDs) [20]. Nevertheless, the function or mechanisms of APOL1 in cancer remain unclear.

In a previous study, we found a new phenomenon called tumor “slimming” in which abnormal lipid accumulation is consumed, which represses the progression of ccRCC [21, 22]. We explained that depletion of lipid droplets can inhibit the development of kidney cancer, but how lipid droplets are produced in kidney cancer and contribute to tumor development remains a mystery.

In this study, we found that HIF2α-dependent APOL1 expression promoted lipid storage, proliferation, and metastasis of ccRCC in vitro and in vivo. APOL1-dependent lipid storage can sustain ER homeostasis. We identified a lncRNA, LINC02609, which was associated with the expression of HIF2α. We found that HIF2α could not only regulate the expression of APOL1 directly by binding to the promoter of APOL1 but also indirectly through the HIF2α/LINC02609/miR-149-5p axis, and play a significant role in lipid metabolism. Thus, our study reveals the previously unrecognized molecular mechanism regulating lipid homeostasis in ccRCC and provides a promising new approach for ccRCC therapy.

## Materials and methods

### Clinical sample preparation

Forty pairs of human ccRCC tissues and adjacent normal tissues were obtained from the Department of Urology, Union Hospital, Huazhong University of Science and Technology during 2016–2017. Forty-four pairs of human ccRCC tissues and adjacent normal tissues were obtained from the Department of Urology, The First Affiliated Hospital of Anhui Medical University during 2021–2022. They were at least 5 cm away from the tumor site in adjacent normal tissues. Tissue specimens were snap frozen in liquid nitrogen before DNA, RNA and protein extraction. The study protocol was approved by the ethics committee of Huazhong University of Science and Technology and The First Affiliated Hospital of Anhui Medical University.

### Cell culture

The human renal cancer cell Lines 786-O, A498, ACHN, Caki-1, OS-RC-2, and the immortalized human proximal renal tubule epithelial cell line HK-2 were purchased from The American Type Culture Collection (ATCC, USA). The RCC cell line SN12-PM6 was supplied by Dr. I.J. Fidler (MD Anderson Cancer Center, Houston, TX). All cells were cultured in Dulbecco’s modified Eagle’s media plus 10% fetal bovine serum (FBS) at 37 °C in 5% CO_2_.

### Oligonucleotide, lentivirus, plasmid and shRNA

Oligonucleotides (miRNA mimics, negative control of mimics, miRNA mutants) were ordered from RiboBio (China). Cells were seeded into plate wells and incubated overnight, and then small RNA molecules were transported into cells by using X-tremeGENE (Roche). The expression lentivirus for APOL1 and the corresponding control vector were all purchased from Genechem, China. Gene-specific shRNA target sequences were synthesized, cloned and inserted into the *Hpa*I and *Xho*I sites of the pSicoR plasmid (Addgene, #11,597). The paired primers were annealed and ligated into pSicoR cut with *Hpa*I and *Xho*I to create shRNA plasmids. The APOL1 3’UTR and LINC02609 3’UTR were separately cloned and inserted into the XhoI-NotI site of the dual luciferase Psicheck2 plasmid (Promega). Human APOL1 promoter sequence cDNAs were PCR amplified using the primers listed in the supplementary, digested by *KpnI* and *Xho*I, and ligated into pGL4.10 respectively. 923-4-mu and the plasmid including the LINC02609 promoter wild-type or mutant sequence were constructed by TSINGKE Biological Technology, China. The expression lentivirus for APOL1 (GV492, Ubi-MCS-3FLAG-CBh-gcGFP-IRES-puromycin) and the corresponding control vector were all purchased from Genechem, China. All plasmids were verified by sequencing. The primers for making these constructs are provided in Supplementary Table S8.

### Quantitative real-time PCR (RT-qPCR), ChIP-seq and chromatin immunoprecipitation (ChIP) assays

Total RNAs was extracted by TRIzol (Invitrogen) and cDNAs was synthesized using a Rever Ace qPCR RT Kit (TOYOBO). Real-time PCR was performed using SYBR Green, Real-time PCR Master Mix (Roche) and the ABI ViiA7 QPCR System (Applied Biosystems). ChIP-seq datasets were downloaded from the NCBI SRA website (https://www.ncbi.nlm.nih.gov/Traces/study/?acc=SRP385097&o=acc_s%3Aa). SRR15838293, SRR15838297, and SRR15838302 were designed to be the input groups, and the experimental group was SRR15838294, SRR15838298, and SRR15838303. All the raw reads were first quality-checked with FastQC 0.11.9 and filtered with trim_galore 0.6.9 (-q 20 –phred 33 –length 20 -e 0.1 -j 4 –stringency 5). Then, the sequences were aligned to the human genome (hg38 assembly) using Bowtie2 and sorted with samtools 1.6. After that, PCR replacements were removed using samtools 1.6. Peaks were then called with MACS2 2.1.0 [23] (--nomodel --extsize 300). Data visualization was performed with IGV 2.11.9 software and the ChIPseeker R package 1.32.1 [24]. ChIP assays were performed with a SimpleChIP® Kit (Agarose Beads) (CST, 22,188 S, Boston, USA) according to the manufacturer’s instructions.

### Luciferase assays

Briefly, 786-O and A498 cells were seeded in 96-well plates (5000 cells per well) and co-transfected with 100 ng psicheck2 Luciferase vector containing target genes 3’UTR with 100 nM or 200 nM miR-149-5p mimics or mutant mimics or negative control of mimics (NC). Forty-eight hours after transfection, Dual- Luciferase Reporter Assay (Promega) was performed according to the manufacturer’s instructions.

### Western blot and antibodies

Western Blot assays were performed as described previously [22]. HIF2α (Novus, #NB100-122,1:1000), APOL1 (abcam, ab108315, 1:1000, GAPDH (Proteintech, 60004-1-Ig, 1:10000), PERK Cell Signaling Technology, #5683, 1:1000, p-PERK abcam, ab192991,1:1000, IRE1α Cell Signaling Technology, #3294, 1:1000, p-IRE1α Novus, #NB100-2323,1:1000, ATF6 Cell Signaling Technology, #65,880, 1:1000.

### Colony formation, cell proliferation, cell migration and invasion

Colony formation were measured two weeks after seeding 1000 cells per well in 6-well plates. Cell proliferation was estimated using the 3-(4,5-dimethylthiazol-2-yl)-5-(3-carboxymethoxyphenyl)-2-(4-sulfophenyl)-2 H-tetrazolium, inner salt (MTS) method (Sigma, USA) according to the manufacturer’s instructions [25].Migration and invasion assays were performed using uncoated and Matrigel™ coated Transwell® inserts according to the manufacturer’s instructions. All experiments were performed in triplicate [26].

### Whole transcriptome sequencing

Total RNA was extracted using the TRIzol (Ambion) following the manufacturer’s protocol. RNA integrity was evaluated using an Agilent 2100 Bioanalyzer (Agilent Technologies, Santa Clara, CA, USA). The samples with an RNA integrity number (RIN) ≥ 7 were subjected to subsequent analysis. The libraries were constructed using TruSeq Stranded mRNA LTSample Prep Kit (Illumina, San Diego, CA, USA) according to the manufacturer’s instructions. Then, these libraries were sequenced on the Illumina sequencing platform (HiSeq 2500 or Illumina HiSeq X Ten) and 125 bp/150 bp paired-end reads were generated. Techniques and methods for whole transcriptome sequencing were provided by Oebiotech, China [22].

### RNA-FISH

Cy3-labeled LINC02609 and DAPI-labeled U6 probes were obtained from RiboBio (Guangzhou, China). RNA FISH was performed using a fluorescent in situ hybridization kit (RiboBio) following the manufacturer’s instructions.

RACE.

The 5′- and 3′-RACE assays were used to explore the termination and initiation site of LINC02609 transcription by 3’-Full RACE Core Set with PrimeScript Rtase (Code No.6106), 5’-Full RACE SMARTer® RACE 5’/3’Kit (Cat. No. 634,860) according to the manufacturer’s protocol in 786-O cell lines.

### Xenograft subcutaneously and tail intravenous injection

Tumorigenesis in nude mice was determined as described previously [26]. Each mice was injected subcutaneously with prepared cells at a single site. A total of 5 × 10^6^ cells were injected subcutaneously into 4 to 5 week-old male nude mice purchased from Vital River Laboratory Animal Technology Co. Ltd. Tumor onset was measured with calipers at the site of injection weekly at different times on the same day. Tumor volume was calculated using the formula, V = 0.5ab^2^, where a represents the larger and b represents the smaller of the two perpendicular indexes. Nude mouse tail vein metastasis model was used to assess the metastatic ability of the tumor cells.786-O cells were stably infected with HIF2α shRNA (versus the negative control), LINC02609 shRNA (versus the negative control) and APOL1 (versus the negative control). Treated cells (1 × 10^6^) were suspended in 150 µl of phosphate-buffered saline and injected intravenously via the tail vein. Mice were killed and livers were resected 30 days after injection. All experiments were approved by the Animal Care and Use Committee of Tongji Medical College of Huazhong University of Science and Technology.

### Immunohistochemistry and immunofluorescence staining

Renal cancer tissue microarrays (HKidE 180Su02-M-046 RB-H-20-B21 and HKidE 180Su02-M-046 RB-H-20-B22) were obtained from Shanghai Outdo Biotech (Shanghai, China). Immunohistochemistry was performed as described previously [22] (HIF2α, abcam, ab199,1:150)(APOL1, Abcam, ab108315,1:150). The tissue array sections were counterstained with hematoxylin. Images were taken with a microscope. The mean proportion of stained cells per specimen was determined semi-quantitatively and scored as follows: 0 for staining 0–1%, 1 for 1–25%, 2 for 26–50%, 3 for 51–75%, and 4 for 75% of the examined cells. Staining intensity was graded as follows: 0, negative staining; 1, weak staining; 2, moderate staining; and 3, strong staining. The histological score (H-score) for each specimen was computed by the formula: H-score = Proportion score* Intensity score. Overall scores of < 8 and ≥ 8 were defined as negative and positive, respectively.

### ER Tracker

ER-Tracker Red was purchased from Beyotime Biotechnology co. Ltd. ER Tracker imaging was performed using ER-Tracker Red KIT (Beyotime, C1041) following the manufacturer’s instructions.

### Statistical analysis

All statistical analyzes were carried out using SPSS 18.0 statistical software. Continuous data were compared using Student’s 2-tailed t test. Data are represented as the mean ± SEM. In all cases, p < 0.05 was considered statistically significant. *p < 0.05; **p < 0.01; ***p < 0.001.

## Results

### Lipid accumulation in ccRCC cells is VHL/HIF-2α dependent, and downstream molecules of this signaling pathway, APOL1 was upregulated and predicted poor prognosis in ccRCC

**Fig. 1:**
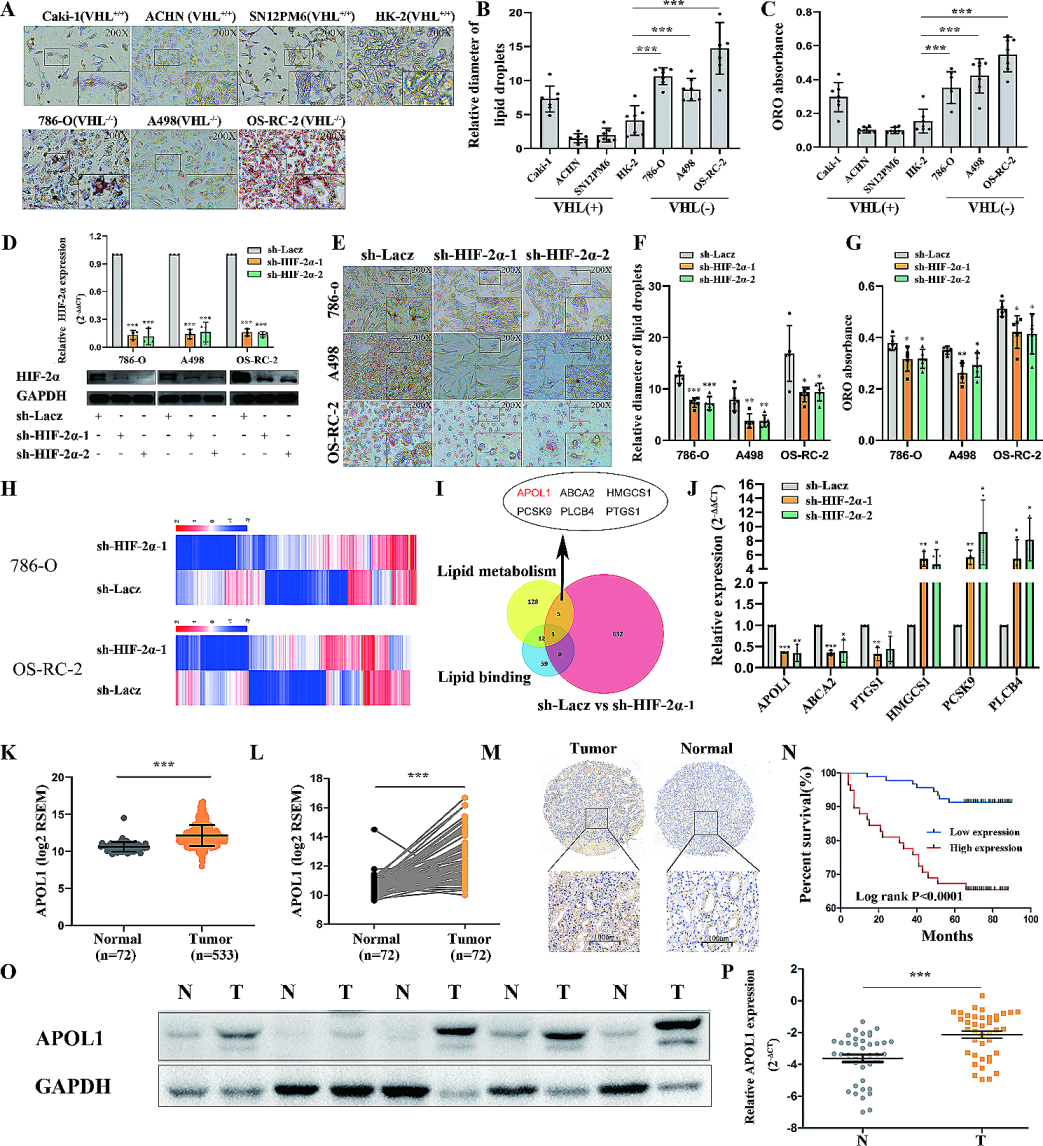
Lipid accumulation in ccRCC cells is VHL/HIF-2α dependent and downstream molecules of this signal pathway-APOL1 was upregulated and predicted poor prognosis in ccRCC. (**A**) Oil red O staining of immortalized human renal tubule epithelial cell line HK-2 and indicated renal carcinoma cell lines (Magnification: 200× & 400×). (**B**) Relative diameter of lipid droplets in indicated cell lines. The data are presented as the means ± SEM. p values of two-tailed Student’s t tests are displayed. (**C**) Quantification of ORO in indicated cell lines. The data are presented as the means ± SEM. p values of two-tailed Student’s t tests are displayed. (**D**) Quantitative real-time PCR and Western blot detected the interference efficiency of HIF2α in ccRCC. (**E**) Photomicrographs of Oil Red O-stained VHL^(−/−)^ cells 786-O, A498 and OS-RC-2 with HIF2α knockdown (Magnification: 200× & 400×). (**F**) Relative diameter of lipid droplets in indicated cell lines. The data are presented as the means ± SEM. p values of two-tailed Student’s t tests are displayed. (**G**) Quantification of ORO in indicated cell lines. The data are presented as the means ± SEM. p values of two-tailed Student’s t tests are displayed. (**H**) The heat-map of cluster analysis of mRNAs based on sequencing results of VHL^(−/−)^ cells 786-O and OS-RC-2 with HIF2α knockdown. (**I**) A Venn diagram of lipid metabolism gene sets, lipid binding gene sets from the Oncomine database (https://www.oncomine.org) and the differentially expressed gene in both 786-O and OS-RC-2 with HIF2α knockdown. (**J**) Q-RT-PCR analysis of HIF2α related mRNAs in renal cancer cell 786-O with HIF2α knockdown. (**K**) The expression of Apol1 in ccRCC (n = 533) and adjacent normal kidney (n = 72). The data were downloaded from the TCGA-KIRC dataset. (**L**) Relative expression of Apol1 in 72 pairs of ccRCC tumor tissues and their corresponding adjacent non-cancerous tissues. The data were downloaded from the TCGA-KIRC dataset. (**M**) Representative images of APOL1 expression in ccRCC and adjacent normal kidney by IHC. (**N**) Kaplan-Meier curve showing overall survival of kidney cancer patients with high or low Apol1 expression (p < 0.0001 by log-rank test). (**O**) The expression of APOL1 protein in cancer is higher than that in the adjacent tissues by Western blot (WB). (**P**) The expression of APOL1 mRNA in cancer is higher than that in the adjacent tissues by q-RT-PCR

To dissect the mechanism of lipid deposition in renal cancer cells, we first evaluated the ability of a panel of three VHL ^(−/−)^ cancerous (786-O, A498 and OS-RC-2), three VHL ^(+/+)^ cancerous (ACHN, Caki-1 and SN12PM6) and one non-tumorigenic (HK-2) cell lines to make lipid droplets (Fig. 1A) [27]. Both quantification of the lipids after Oil Red O extraction normalized to the cell number and detection of the relative diameter of lipid droplets showed that VHL ^(−/−)^ cancerous (786-O, A498 and OS-RC-2) cells stained more Oil Red O than non-tumorigenic VHL ^(+/+)^ (HK-2) and VHL ^(+/+)^ cancerous (ACHN, Caki-1 and SN12PM6) cells (Fig. 1B-C and Supplementary Fig. S1A, B). HIF1α and HIF2α are broadly expressed in many human cancers and are the most important downstream biomolecules of VHL [9]. However, we observed that the expression of HIF1α in tumor tissues was significantly lower than that in adjacent tissues in TCGA (Supplementary Fig. S2A and S2B). Chuan Shen et al. also found that HIF1α had the credentials of a kidney cancer suppressor gene [10]. For these reasons, we mainly focused on HIF2α in this research. Supplementary Fig. S2C and S2D show that the messenger RNA (mRNA) levels of HIF2α were high in tumors in renal cancer. HIF-2α has been implicated in angiogenesis, immuno-evasion and multiple other processes in ccRCC. In VHL-defective RCC cells, Raju et al. demonstrate that the protumorigenic genes encoding cyclin D1, transforming growth factor alpha, and vascular endothelial growth factor respond specifically to HIF-2 [28]. Yosra Messai et al. provide insight into the link between VHL mutations, the HIF-2α-related pathway, and PD-L1 expression, and point to a critical role of VHL/HIF-2α axis in controlling anti PD-1/PD-L1 response [29]. Bo Qiu et al. demonstrate that HIF2α promotes lipid storage and cell viability in ccRCC via upregulation PLIN2 [30]. To further study the role of HIF2α in RCC, we examined its clinical relevance in cancer patients. We analyzed HIF2α expression in a tissue microarray including 150 ccRCC tissues and 30 adjacent normal tissues by immunohistochemistry (Supplementary Fig. S2E) (Supplementary Table S1A) and found that the HIF2α-positive group showed significantly poorer overall survival than the HIF2α-negative group (Fig. S2F) (Supplementary Table S1A), indicating that HIF2α may be a potentially valuable biomarker for the prognosis of ccRCC. To explore the role of HIF2α in renal cancer cells, we stably inhibited HIF2α in three VHL^(−/−)^ ccRCC cell lines (786-O, A498 and OS-RC-2) with lentiviruses carrying shRNA for HIF2α and a control nonspecific shRNA (LacZ) (Fig. 1D). Oil red O staining was used as a visual indicator of intracellular lipids in ccRCC (Fig. 1E). The results showed that there was an obvious lipid reduction in HIF2α depleted renal cancer cells (Fig. 1F and G). Recent studies have shown that HIF2α/PLIN2 promotes lipid storage and tumor growth in ccRCC in vivo and in vitro [30]. However, how HIF2α regulates relevant molecules and affects the lipid metabolism and lipid droplet accumulation remains unknown. In this study, we chose a RNA-seq in VHL^(−/−)^ ccRCC cell line 786-O and OS-RC-2 carrying shRNA for HIF2α or LacZ (Fig. 1H). We concluded that 1778 mRNA transcripts were upregulated and 1808 mRNA transcripts were downregulated in renal cancer cell line 786-O (sh-HIF2α vs. sh-LacZ). We also found that 1679 mRNA transcripts were up-regulated and 1794 mRNA transcripts were down-regulated in the renal cancer cell line OS-RC-2 (sh-HIF2α vs. sh-LacZ) (Supplementary Table S2). In the integrated analysis, there were 509 upregulated mRNAs and 546 downregulated mRNAs in both the renal cell lines 786-O (sh-HIF2α vs. sh-LacZ) and OS-RC-2 (sh-HIF2α vs. sh-LacZ) (Fig. 1H). A Venn diagram showed that there were six genes (APOL1, ABCA2, HMGCS1, PCSK9, PLCB4 and PTGS1) involved in lipid metabolism, lipid binding and consistent differential expression in both 786-O (sh-HIF2α vs. sh-LacZ) and OS-RC-2 (sh-HIF2α vs. sh-LacZ) (Fig. 1I). Further q-RT-PCR reach a similar conclusion (Fig. 1J). The expression and prognosis of these six genes were detected in the TCGA database, and among the six genes, only two genes’ expression and prognosis were consistent. (It is highly expressed in tumors, and the higher the expression, the worse the prognosis or low expression in tumors, and the lower the expression, the better the prognosis) (Supplementary Fig. S3). APOL1 is highly expressed in renal tumors and is associated with poorer prognosis, whereas HMGCS1 is expressed at low levels in renal tumors and is associated with better prognosis. Cholesterol and esterified cholesterol are the most prominent lipids stored in ccRCC, accumulating higher levels than in compared to normal kidney tissue [31, 32]. HMGCS1 encodes rate-limiting enzymes in the *de novo* cholesterol biosynthetic pathway but is expressed at low levels in renal tumors. We researched the NCBI GEO repository and found that knockdown of HIF2α downregulated the expression of APOL1 in A498 cell lines in the microarray analysis (Supplementary Fig. S4) [33].Therefore, in this study, we mainly focused on APOL1. We found that APOL1 was expressed at a higher level in ccRCC in TCGA (Fig. 1K and L) and other databases (Supplementary Fig. S5A). Additional results revealed that APOL1 levels were remarkably correlated with, TNM, grade, stage, metastasis and recurrence in ccRCC (Supplementary Fig. S5B) (Table 1). To further study the role of APOL1, we examined its clinical relevance in cancer patients at the protein level. We analyzed APOL1 expression in 150 ccRCC samples by immunohistochemistry and found that high APOL1 protein expression was significantly correlated with the tumor (T) and stage (Supplementary Fig. S5C and D). Moreover, the APOL1-positive group showed obviously poorer overall survival than the APOL1-negative group (Fig. 1M and N). To further support this conclusion, we examined APOL1 protein expression in 5 renal cancer tissues and their corresponding noncancerous tissues from our laboratory and found APOL1 expression was higher in cancer tissues than in adjacent normal tissues (Fig. 1O). We also examined the expression of APOL1 mRNA in 40 renal cancer tissues and their corresponding noncancerous tissues form The Union hospital and obtained similar results (Fig. 1P)( Supplementary Table S1B). The renal tissues form The First Affiliated Hospital of Anhui Medical University got similar results (Supplementary Fig. S5E) (Supplementary Table S1C). Above all, these results confirmed that high APOL1 expression was associated with poor prognosis, and that overexpressed APOL1 might be crucial in ccRCC tumorigenesis and progression.

**Table 1:** The characteristic of APOL1 in clear cell renal cell carcinoma

Characteristic		Total (n = 514)	APOL1		p Value
			Low(257)	High(257)	
Gender	Male	179	107	72	
	Female	335	150	185	0.001
Age	<=60	255	123	132	
	> 60	259	134	125	0.427
T	T1&T2	330	184	144	
	T3&T4	184	71	113	0.000
N	N0	234	117	117	
	N1	16	4	12	0.052^a,c^
	Nx	264	136	128	0.120
M	M0	419	219	200	
	M1	73	23	50	0.001
	Mx	22	15	7	0.001
Stage	1,2	314	180	134	
	3,4	200	77	123	0.000
Grade	1,2	198	144	95	
	3,4	245	108	162	0.000^b^
	X	5	5	0	0.000^c^
Recurrence	No	405	212	193	
	Yes	109	45	64	0.040

a: N0,N1 and Apol1 expression

b: Grade1,2 Grade3,4 and Apol1 expression

c: Yates’s correction for continuity

### APOL1 repression controls tumor progression and lipid deposition in VHL^(−/−)^ ccRCC cells

**Fig. 2:**
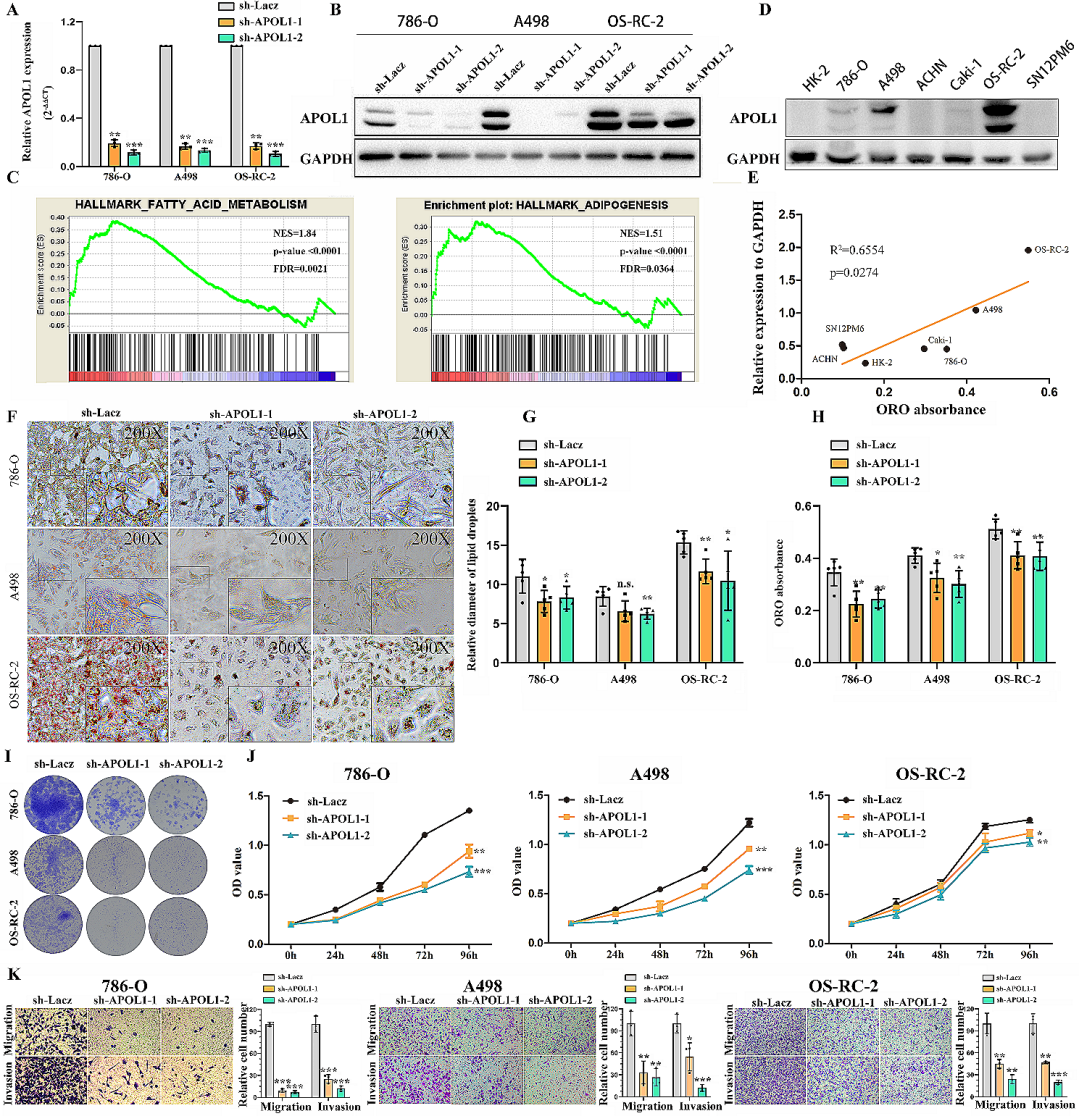
APOL1 repression controls tumor progression and lipid deposition in VHL^(−/−)^ ccRCC cells. (**A**, **B**) VHL^(−/−)^ ccRCC (786-O, A498 and OS-RC-2) were transfected with two independent shRNAs against APOL1 or a control (Lacz). qRT-PCR and Western blot analysis of APOL1 are shown. (**C**) GSEA assays for the correlation of fatty acid metabolism, adipogenesis and mRNA levels of APOL1 according to the TCGA database. FDR < 25%, p < 0.05 was considered statistically significant. (**D**) APOL1 protein levels were detected by immunoblot analysis in renal cancer cell lines. GAPDH served as an internal control. (**E**) The correlation between APOL1 protein and the quantification of ORO in renal cancer cell lines and HK-2 cell line. (**F**) Photomicrographs of Oil Red O-stained VHL^(−/−)^ cells 786-O, A498 and OS-RC-2 with APOL1 knockdown (Magnification: 200× & 400×). (**G, H**) Quantification of ORO, relative diameter of lipid droplets in indicated cell lines. The data are presented as the means ± SEM. p values of two-tailed Student’s t tests are displayed. (**I**) Representative micrographs of crystal violet-stained cell colonies analyzed by clonogenic formation. (**J**) MTS assays revealed cell growth curves of indicated cells. (**K**) Migration and invasion assays for indicated renal cancer cells. Representative photographs were taken at 200× magnifcation; the number of migrated cells was quantified in three random images from each treatment group

To further study the effect of APOL1 on lipid metabolism in ccRCC, we stably inhibited APOL1 in three VHL^(−/−)^ ccRCC cell lines (786-O, A498 and OS-RC-2) with lenti-viruses carrying shRNA (Fig. 2A and B). To identify APOL1-associated biological signaling pathways on an unbiased basis, we performed Gene Set Enrichment Analysis (GSEA) using high throughput RNA-sequencing data of the TCGA cohort. APOL1 expression was used as the phenotype label. Among all the predefined Hallmark gene sets, fatty acid metabolism and adipogenesis signaling pathway were found to be significantly associated with APOL1 expression in the TCGA cohort (Fig. 2C), suggesting that APOL1 may be highly associated with fatty acid metabolism and adipogenesis, both of which were related to lipid metabolism according to the data from TCGA database. We then evaluated the expression of APOL1 in three VHL ^(−/−)^ cancerous (786-O, A498 and OS-RC-2), three VHL ^(+/+)^ cancerous (ACHN, Caki-1 and SN12PM6) and one non-tumorigenic (HK-2) cell lines and found that VHL ^(−/−)^ cells have higher APOL1 expression than VHL ^(+/+)^ cancerous and HK-2 (Fig. 2D) cells, which is highly consistent with lipid droplet expression in the indicated cell lines. Further analysis showed that there was a positive correlation between APOL1 protein and the quantification of ORO (Oil Red O) in renal cancer cell lines and the HK-2 cell line (Fig. 2E). Both GSEA and correlation analysis showed that APOL1 was related to lipid deposition. ORO staining showed that there was an obvious lipid reduction in APOL1 deficient renal cancer cells (Fig. 2F-H). Colony formation assays (Fig. 2I) and MTS assays (Fig. 2J) revealed that APOL1 knockdown inhibited cell proliferation in 786-O, A498 and OS-RC-2 cells. Quantification of apoptosis by Annexin V/PI double labeling indicated that a remarkably higher apoptotic index was detected in sh-APOL1-1 and sh-APOL1-2 transfectants relative to control cells in 786-O, A498 and OS-RC-2 cells (Supplementary Fig. S6). Further transwell assays suggested that APOL1 knockdown suppressed renal cancer cell migration and invasion (Fig. 2K). ORO staining showed that there was little lipid reduction in APOL1 deficient ACHN, a VHL (+/+) renal cancerous cell lines. Colony formation, MTS and transwell assay revealed that knockdown APOL1 inhibited cell proliferation and metastasis in ACHN cells, but not as pronounced as the VHL (-/-) cancerous (786-O, A498 and OS-RC-2) (data not shown). These results demonstrated that APOL1 can promote lipid deposition and tumor progression in VHL^(−/−)^ ccRCC.

### Transcriptional regulation of APOL1 expression by HIF-2α in ccRCC cells

**Fig. 3:**
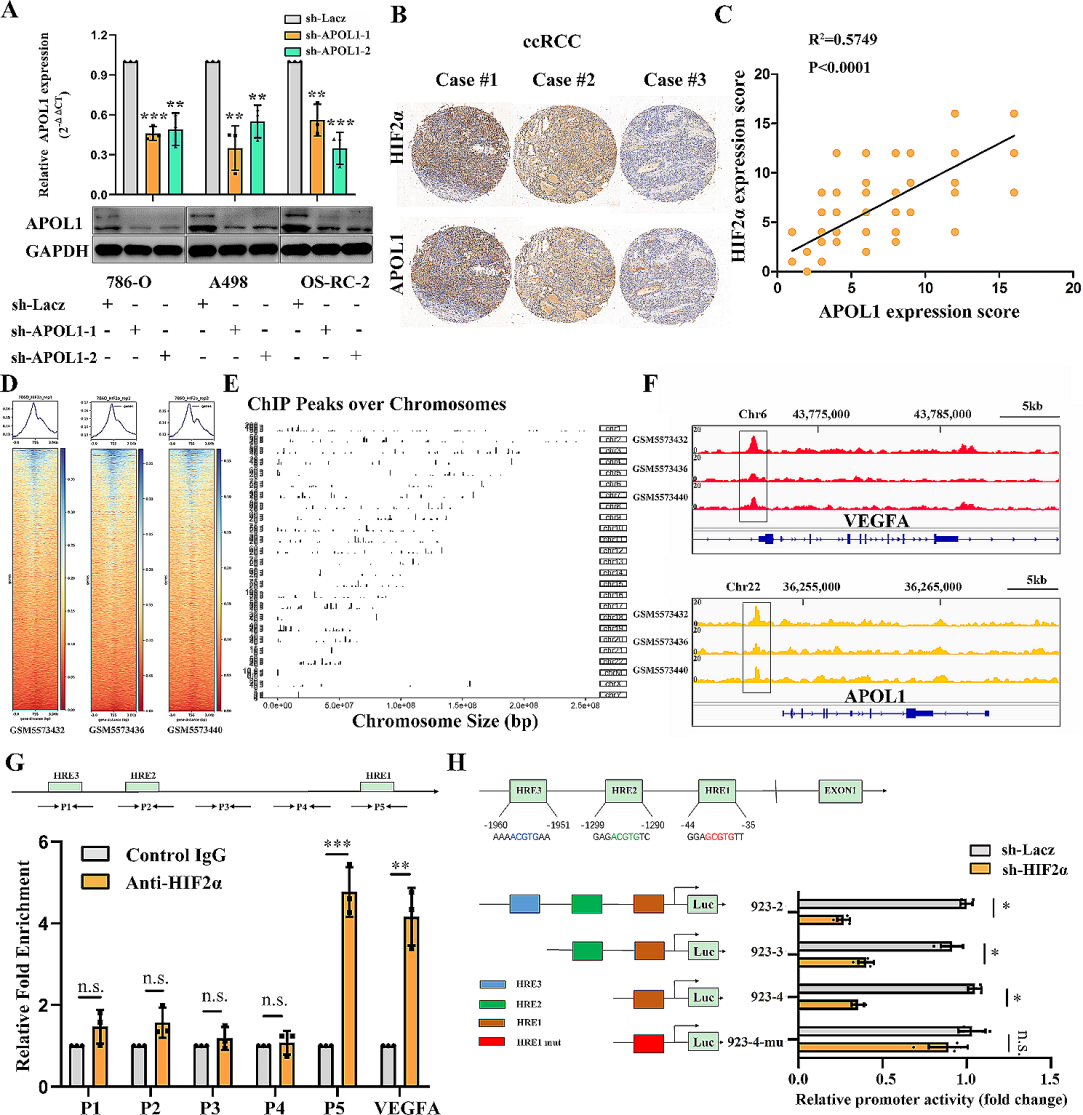
Direct binding of HIF-2α to the APOL1 promoter. (**A**) Quantitative real-time PCR and Western blot detected the interference efficiency of APOL1 in indicated cell lines. (**B**) Representative images of HIF-2α and APOL1 expression in ccRCC serially cut tissue by IHC. (**C**) Correlation between the expression of HIF2α protein and APOL1 protein in clear cell renal cell carcinoma (n = 150). (**D**) The reads distributed on two sides of the transcription start site (TSS). (**E**) ChIP peaks over chromosomes by ChIP-seq technology employing the primary antibody against HIF-2α (GSM5573436). (**F**) ChIP-seq enrichment profiles of HIF-2α in genomic region spanning the VEGFA (positive control) and APOL1. (**G**) Diagram of the APOL1 promoter region analyzed for putative HREs (green boxes) from the − 2000 to the transcriptional start site of APOL1 (+ 1). Three putative HREs located at different sites in the APOL1 promoter sequence. Primer pairs used for PCR amplification after ChIP are indicated. Primer 1 pairs (P1) amplified product including HRE3, Primer 2 pairs (P2) amplified product including HRE2, Primer 5 pairs (P5) amplified product including HRE1. Results of ChIP-real-time PCR and ChIP-PCR assay conducted using chromatins isolated from 786-O cells. A specific anti- HIF-2α antibody was used, and normal IgG was used as a control. 2% of the total cell lysates were subjected to PCR before immunoprecipitation (input control). The experiments were performed three times independently. (**H)** The fragment including putative HRE were structured into pGL4.10 plasmid (pGL4.10-923-2 including putative HRE3, HRE2 and HRE1, pGL4.10-923-3 including putative HRE2 and HRE1, pGL4.10-923-4 including putative HRE1, pGL4.10-923-4-mu including the mutant HRE1). APOL1 promoter reporters (pGL4.10-923-2, 923-3, 923-4 and 923-4 mutant) and pGL4.73 were co-transfected into 239T cells for 24 h. The APOL1 promoter activity was then examined using a dual luciferase assay kit

Further study showed that HIF2α could positively regulate the expression of APOL1 by WB and q-RT-PCR in 786-O, A498 and OS-RC-2 cells (Fig. 3A). Then, we found that APOL1 correlates with HIF2α in human normal renal tissue from the TCGA Data Portal (R^2^ = 0.1414, p = 0.0011) (Supplementary Fig. S7). Tissue microarrays of APOL1 and HIF2α were derived from serially cut tissue (Fig. 3B) and statistical analysis revealed that the expression of APOL1 was positively correlated with HIF2α expression (R^2^ = 0.5749, p < 0.0001) (Fig. 3C). Xavier Darzacq et al confirmed intrinsically disordered region dependent binding and activation of a specific subset of HIF target genes by CHIP-seq [34]. We then thoroughly analyzed the genome-wide target sites of HIF-2α in 786-O RCC cells by using the ChIP-seq raw data [34] (Fig. 3D). The peaks over chromosomes indicated different peak values. The abscissa shows the chromosome size, the right ordinate represents the chromosome number, and the left ordinate represents each chromosome peak value (Fig. 3E). Multiple HIF2α binding events occur in various intronic regions containing HREs such as VEGFA, GLUT1 and so on. In the ChIP-seq data, a large number of peaks that can bind to the HIF2α anti-body were enriched in the VEGFA and APOL1 promoter regions in 786-O cells (Fig. 3F). To continue to explore the mechanism of regulation of APOL1 expression by HIF-2α, we analyzed the APOL1 promoter sequence 5’-A/GCGTG-3’ for potential HREs, which were described previously [28, 35]. Sequence analysis of the APOL1 promoter revealed three putative HREs located at -21 bp (HRE1), -1276 bp (HRE2), and − 1937 bp (HRE3) relative to the transcriptional start site of APOL1 (Fig. 3G). To determine whether HIF-2α regulates APOL1 expression at the transcriptional level, we conducted a ChIP assay using chromatin prepared from 786-O cells. The results confirmed that HIF-2α directly bound to the HRE1 site (Fig. 3G) rather than other putative HRE sites in the APOL1 promoter. VEGFA was used as a positive control. To provide more evidence that HIF-2α binds directly to the APOL1 promoter, a luciferase reporter assay was performed. We generated the full-length APOL1 promoter (including HRE3, HRE2 and HRE1) pGL4.10-923-2 and deletion APOL1 promoter (including HRE2 and HRE1) pGL4.10-923-3, deletion APOL1 promoter (including HRE1) pGL4.10-923-4 and mutant APOL1 promoter (including HRE1 mutant) of it (Fig. 3H). Then, we co-transfected the full-length, deletion or mutant reporters with pGL4.73 vectors into 293T cells. Luciferase reporter assay results demonstrated that a mutant HRE1 site, markedly reduced the promoter activity of APOL1 induced by HIF-2α (Fig. 3H). These data strongly indicated that HIF-2α bound to the APOL1 promoter and transcriptionally regulated APOL1 expression in 786-O cells.

### Transcriptional regulation of LINC02609 expression by HIF-2α in ccRCC cells

**Fig. 4:**
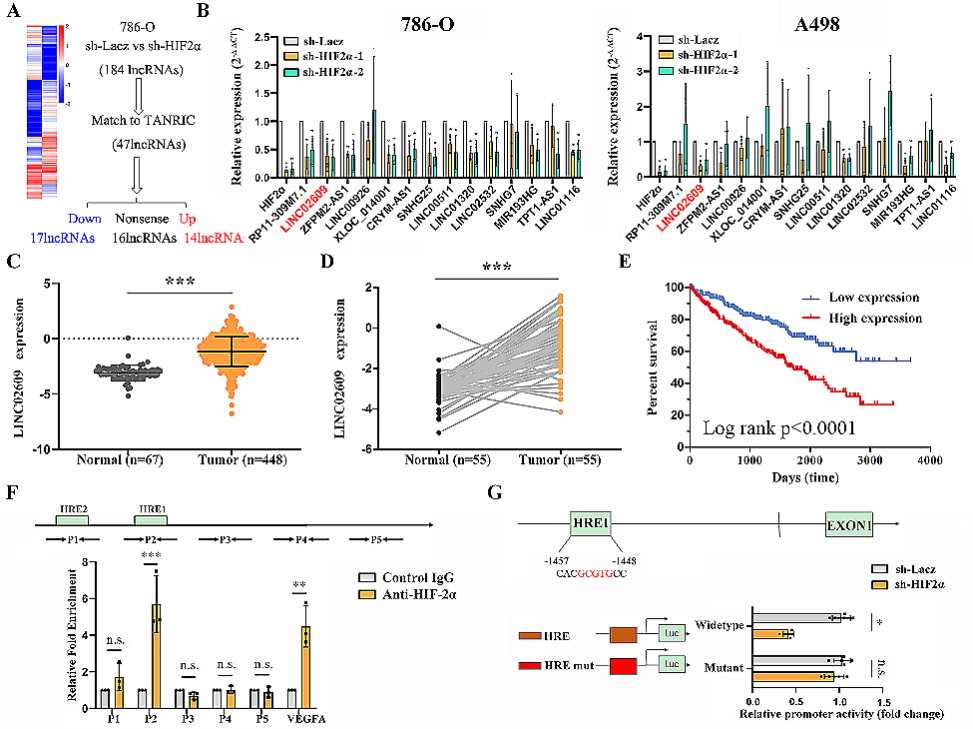
Direct binding of HIF-2α to the LncRNA LINC02609 promoter. (**A**) The heat-map of cluster analysis of lncRNA based on sequencing results of VHL^(−/−)^ cells 786-O with HIF2α knockdown. 184 lncRNA transcripts were down-regulated in HIF2α knockdown cell lines. Match to the TANRIC, 47 lncRNA transcripts were found in the database, among which 14 lncRNA transcripts existed higher expression in cancer than in normal. (**B**) Quantitative real-time PCR detected the 14 lncRNA transcripts in renal cancer cell 786-O and A498 (sh- HIF2α vs. sh-Lacz). (**C**) The expression of LINC02609 in ccRCC (n = 448) and adjacent normal kidney (n = 67). The data were downloaded from the TCGA-KIRC dataset from TANRIC. (**D**) Relative expression of LINC02609 in 55 pairs of ccRCC tumor tissues and their corresponding adjacent non-cancerous tissues. The data were downloaded from the TCGA-KIRC. (**E**) Kaplan-Meier curve showing overall survival of kidney cancer patients with high or low LINC02609 expression (p < 0.0001 by log-rank test). (**F**) ChIP-real-time PCR were conducted using chromatins isolated from 786-O cells. A specific HIF2α antibody was used, and normal IgG was used as a control. 2% of the total cell lysates was subjected to PCR before immunoprecipitation (input control). The experiments were performed three times independently. (**G**) APOL1 promoter reporters (pGL4.10-924 wide-type and 924 mutant) and pGL4.73 were co-transfected into 239T cells for 24 h. The APOL1 promoter activity was then examined using a dual luciferase assay kit

To identify lncRNAs regulated by HIF-2α, we analyzed the expression profiles of lncRNAs in the human renal cancer cell line 786-O (sh-HIF-2α vs. sh-Lacz) using whole transcriptome sequencing. As shown in Fig. 4A and Supplementary Table S3, the RNA-seq analysis identified 176 upregulated and 184 downregulated lncRNAs in the renal cancer cell line 786-O (sh-HIF-2α vs. sh-Lacz). HIF is not typically considered a direct transcriptional repressor [36], so we mainly focused on the 184 downregulated lncRNAs in 786-O cells (sh-HIF-2α vs. sh-Lacz). Jun Li et al. used recent large-scale RNA-seq datasets, especially those from The Cancer Genome Atlas (TCGA), to develop “The Atlas of Noncoding RNAs in Cancer” (TANRIC; http://bioinformatics.mdanderson.org/main/TANRIC:Overview), a user-friendly, open-access web resource for interactive exploration of lncRNAs in cancer [37]. We found that 47 lncRNAs were annotated in the TANRIC among the 184 downregulated lncRNAs and 14 lncRNAs were upregulated and 17 lncRNAs were downregulated in renal cancer tissue compared with adjacent normal tissue matched to TANRIC (Fig. 4A) (Supplementary Table S4). We detected these 14 lncRNAs in 786-O and A498 cell lines, and found that three lncRNAs, including LINC02609, LINC01320 and LICN01116, decreased simultaneously by q-RT-PCR in 786-O and A498 (Fig. 4B). To investigate the effect of LINC02609, LINC01320 and LICN01116 in ccRCC, we down-regulated the expression of LINC02609, LINC01320 and LICN01116 in renal cancer cell lines (786-O and A498). Further study showed that knockdown LINC02609 or LINC01320 suppressed renal cancer cell proliferation and metastasis (data not shown). HIF-2α acts by binding to HRE upon hypoxia or normoxia with VHL mutation, and LINC02609 has two HREs in the promoter. We also found that LINC02609 had higher expression in renal tumors than in normal renal tissue in TCGA from TANRIC (Fig. 4C and D). Additional results revealed that LINC02609 levels were remarkably correlated with TNM, grade, stage and recurrence in ccRCC (Supplementary Table S5). Furthermore, the LINC02609 -positive group showed significantly poorer overall survival than the LINC02609-negative group (Fig. 4E) (Supplementary Fig. S8). In our assessment, the full-length LINC02609 transcript was 716 nt in 786-O, which was examined using the 5′ and 3′ rapid amplification of cDNA end (RACE) method (Supplementary Table S6). To determine whether HIF-2α regulates LINC02609 expression at the transcriptional level, we conducted a ChIP assay using chromatin prepared from 786-O cells. The results confirmed that HIF-2α directly bound to the HRE2 site in the LINC02609 promoter in 786-O cells (Fig. 4F). VEGFA was used as a positive control. A luciferase reporter assay demonstrated that a mutant HRE site, markedly reduced the promoter activity of LINC02609 induced by HIF-2α (Fig. 4G). Collectively, these results indicated that HIF-2α regulates LINC02609 transcriptional activity by binding to its promoter.

### The HIF-2α/LINC02609/miR-149-5p axis regulates APOL1 expression in ccRCC cells

**Fig. 5:**
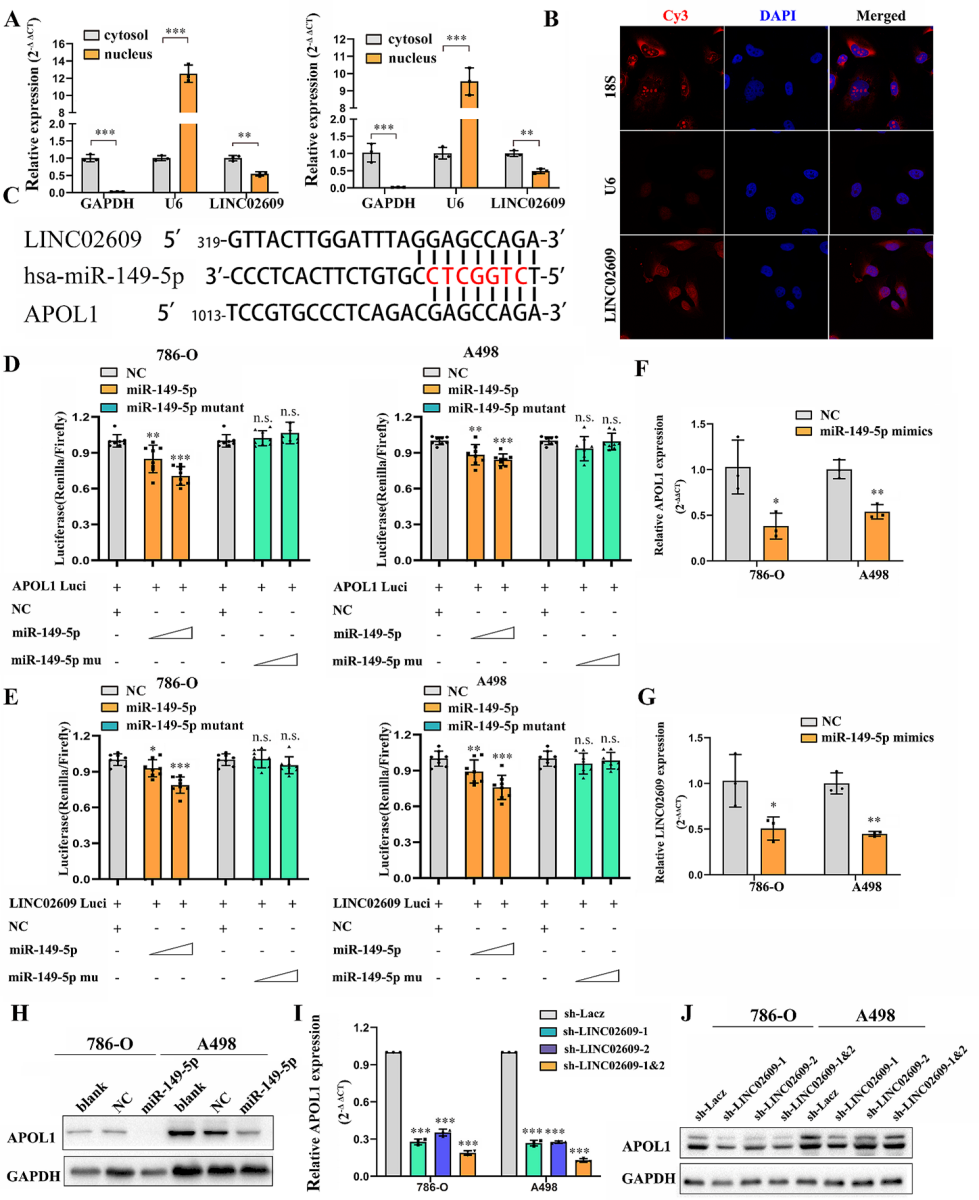
LINC02609 /miR-149-5p regulates Apol1 expression in ccRCC cells. (**A**) Subcellular distribution of LINC02609 in 786-O and A498 cells. GAPDH was used as cytoplasm control and U6 was used as nucleus control. (**B**) RNA fluorescence in situ hybridization (FISH) showed that LINC02609 was predominantly localized in cytoplasm. U6 was mainly localized in nucleus, used as negative control. 18 S was mainly localized in cytoplasm, used as positive control. LINC02609, U6, and 18 S probes were labeled with Cy3, Nuclei was stained with DAPI. (**C**) Schematic miR-149-5p putative target sites in 3′ UTRs of LINC02609 and APOL1. (**D**) Luciferase reporters harboring putative target sites in the 3′ UTRs of APOL1 were co-transfected with 50 and 100 nM of miR-149-5p mimics or miR-149-5p mutant mimics in 786-O and A498 cells. (**E**) Luciferase reporters harboring putative target sites in the 3′ UTRs of LINC02609 were co-transfected with 50 and 100 nM of miR-149-5p mimics or miR-149-5p mutant mimics in 786-O and A498 cells. Relative luciferase activity was plotted as the mean ± SEM of three independent experiments. (**F, G**) Q-RT-PCR analysis of APOL1 and LINC02609 in renal cancer cell 786-O and A498 after transfected with miR-149-5p mimics. (**H**) Western blotting analysis of APOL1 expression in indicated cells. GAPDH was used as a loading control. (**I**) Q-RT-PCR analysis of APOL1 in renal cancer cell 786-O and A498 with LINC02609 knockdown. (**J**) WB analysis of the protein levels of APOL1 in response to deregulated LINC02609 expression of indicated cells

To further analyze the functions and mechanisms of LINC02609 in ccRCC, subcellular fractionation localization assays demonstrated that LINC02609 was mainly localized in the cytoplasm. The cytoplasmic and nuclear ratios were approximately 2:1 in renal cancer cell lines 786-O and A498 (Fig. 5A). We also used RNA-FISH to examine the subcellular localization of LINC02609, Cy3-labeled probes specific for human LINC02609 were used and the analysis confirmed that LINC02609 was localized predominantly in the cell cytoplasm (Fig. 5B). Recently, many RNA transcripts have been reported to function as competing endogenous RNAs (ceRNAs) by competitively binding common microRNAs [25, 38]. MicroRNAs are known to exert their functions mainly, if not exclusively, in the cytoplasm [39]. Then bioinformatics analysis showed that the APOL1 3’UTR and LINC02609 can bind with miR-149-5p (Fig. 5C). Reporter assays showed that the activity of luciferase linked with the 3′ UTR of APOL1 or LINC02609 was repressed in a dose-dependent manner in miR-149-5p mimic–transfected 786-O and A498 cells, compared with control cells. Of note, mutations brought into the seed sequence of miR-149-5p abolished its suppressive effects (Fig. 5D and E). Then, we transfected miR-149-5p mimics, negative controls and mock controls into the indicated cells and detected the expression of APOL1 and LINC02609 in 768-O and A498 cells. The expression of LINC02609 and APOL1 was widely decreased in 768-O and A498 cells transfected with miR-149-5p, as shown by q-RT-PCR (Fig. 5F and G). We obtained a similar result of APOL1 protein expression in 768-O and A498 cells transfected with miR-149-5p by WB (Fig. 5H). Further study showed that knockdown of LINC02609 regulated APOL1 protein expression (Fig. 5I and J). We also found that LINC02609 overexpression trap hsa-miR-149-5p and rescue hsa-miR-149-5p-induced decrease APOL1 expression (Supplementary Fig. S9). Collectively, these data indicated that LINC02609 functions as a competing endogenous RNA to regulate APOL1 expression by sponging miR-149-5p in ccRCC.

### APOL1-dependent lipid storage is required for ER homeostasis in ccRCC

**Fig. 6:**
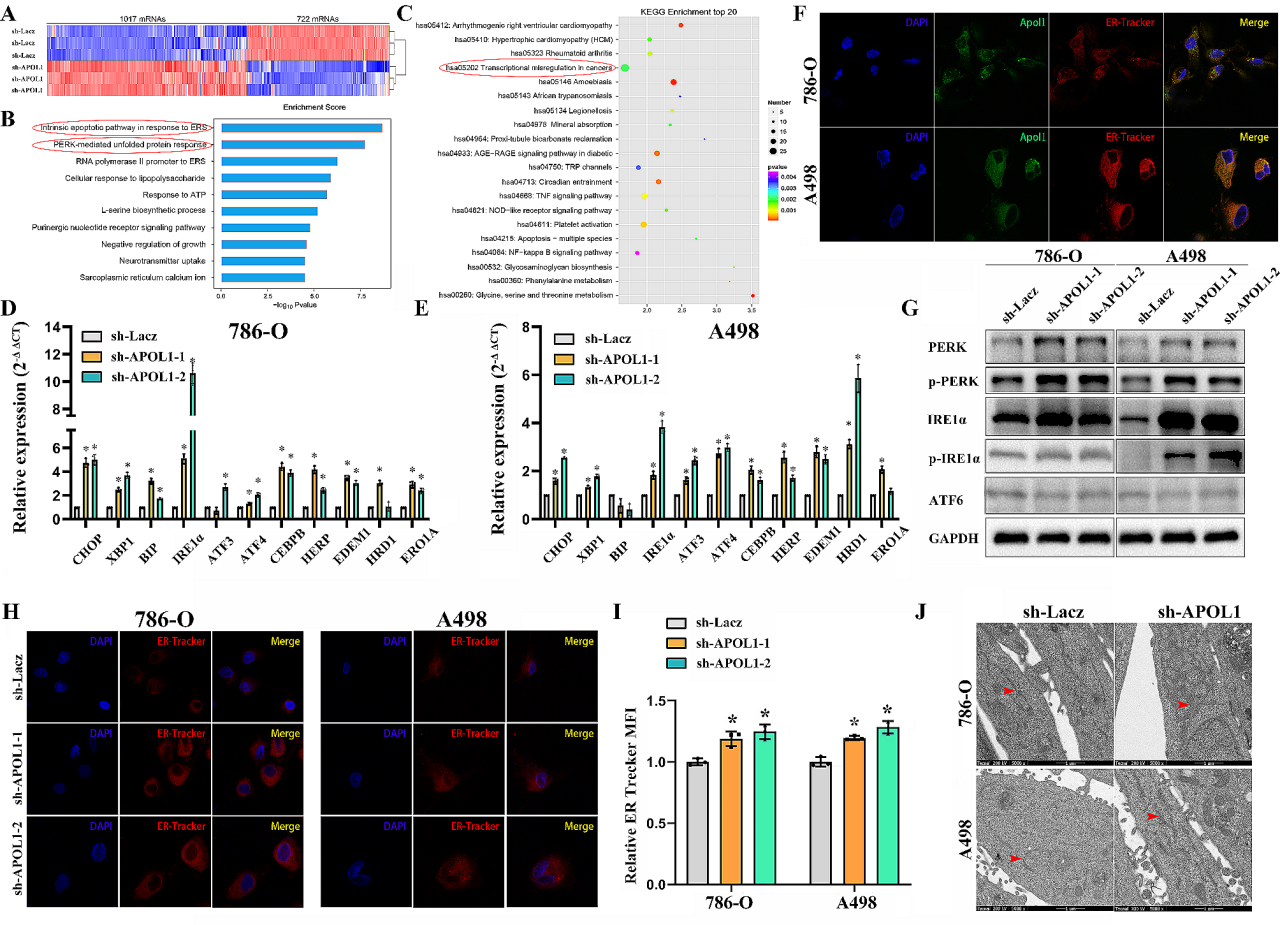
APOL1-dependent lipid storage is required for ER homeostasis and cell viability in ccRCC. (**A**) Heatmap showing the expression change of genes in 786-O cells after transfection of APOL1 shRNA and control shRNA Lacz. Gene expression is shown as RPKM after normalization. (**B**) GO enrichment for the indicated cells based on the results from sequencing. (**C**) KEGG enrichment top 20 for indicated cells based on the results from sequencing. (**D, E**) Q-RT-PCR analysis of UPR target genes in renal cancer cell 786-O and A498 with APOL1 knockdown. (**F**) 786-O and A498 cells were double immunostained with anti-APOL1 antibody (green) and ER-Tracker probe (red). The cell nuclei were counterstained with DAPI (blue). The co-localization between the protein and endoplasmic reticulum is shown in the merge panel (Magnification: 640×). (**G**) Western blot for UPR sensors was performed in 786-O and A498 cells with APOL1 knockdown. (**H, I**) ER-Tracker Red (500 nmol/L) staining of live cells described was performed. Representative images (left) and quantification of ER Tracker fluorescence are shown (right). Fluorescence was normalized to forward scatter for each event to account for differences in cell size (Magnification: 640×). P values were determined by the Students’ t test. (**J**) Transmission electron microscopy (TEM) of control and APOL1-depleted cells is shown. Red arrows, rough ER. (Magnification: 5000×)

Next, we used RNA-seq data from 786-O cells transfected with sh-Lacz or sh-APOL1 to characterize APOL1-mediated changes in gene expression. As shown in Fig. 6A and Supplementary Table S7, RNA-seq analysis identified 1017 upregulated and 722 downregulated mRNAs in the renal cancer cell line 786-O (sh-APOL1 vs. sh-Lacz). Cancer-related genes such as CXCR4, MMP7, CDC20 and CDK6, and unfolded protein response (UPR)-related genes such as ATF3, ATF4, XBP1 and CHOP, showed significant differences in expression in the indicated cells (Supplementary Fig. S10A). Further q-RT-PCR reached a similar conclusion (Supplementary Fig. S10B). These data supported a strong consistency between the qPCR results and RNA-seq data. Additional bioinformatic analyses were utilized to analyze the sequencing results. The results of gene ontology (GO) enrichment analysis showed that the functions of APOL1 were mainly related to the intrinsic apoptotic signaling pathway in response to endoplasmic reticulum stress and the PERK-mediated UPR (Fig. 6B). The endoplasmic reticulum (ER) is an essential organelle for multiple cellular functions, including the biosynthesis of proteins, lipids or sterols and the transport of synthesized proteins and so on. The GO analysis drops a hint that APOL1 related lipid deposition may retain ER homoeostasis. KEGG (Kyoto Encyclopedia of Genes and Genomes) enrichment analysis showed that it was related to African trypanosomiasis which was reported and confirmed. The KEGG analysis also showed that the depletion of APOL1 is related to transcriptional misregulation in cancers, which means that it may have a significant impact on the development of tumors (Fig. 6C).

To verify the RNA-seq data and that the depletion of APOL1 affected endoplasmic reticulum homoeostasis in the renal cancer cell lines 786-O and A498, we detected UPR-related gene and obtained the similar results (Fig. 6D and E). We also found that APOL1 and ER Tracker fluorescence were precisely co-localization in the renal cancer cell lines 786-O and A498 (Fig. 6F). Western blot for UPR sensors was performed in 786-O and A498 cells with APOL1 knockdown. We concluded that knockdown of APOL1 can activate the UPR sensors PERK and IRE1α (Fig. 6G). Previous studies have revealed that hypoxia is often a cause of ER stress and HIF modulates the expression and activity of ER stress sensors [40, 41]. In the present study, we performed a rescue experiment by co-transfecting with HIF2α shRNA (versus the negative control) and APOL1 (versus the negative control) into 786-O and A498 cells. We found that ER homeostasis aggravated by HIF2α and APOL1 can partly reverse the strain by HIF2α shRNA (Supplementary Fig. S11).

Furthermore, ER Tracker imaging indicated ER expansion in APOL1-depleted renal cancer cells 786-O and A498 (Fig. 6H and I), and ultrastructural analysis by transmission electron microscopy (TEM) confirmed the presence of irregularly and dilated rough ER (Fig. 6J), both of which are consistent with ER stress. Microbial-derived tunicamycin (Tm) is the most commonly deployed experimental inducer of ER stress. Tm blocks N-glycosylation and causes misfolding of many proteins in the ER [30, 42]. We have performed MTS assay showed that APOL1-depleted cells were more sensitive to tunicamycin treatment, compared with controls in renal cancer cell lines 786-O (Supplementary Fig. S12).

### APOL1 partly reverses Tumor progression and lipid storage initiated by the HIF2α /LINC02609 axis in vitro and in vivo

**Fig. 7:**
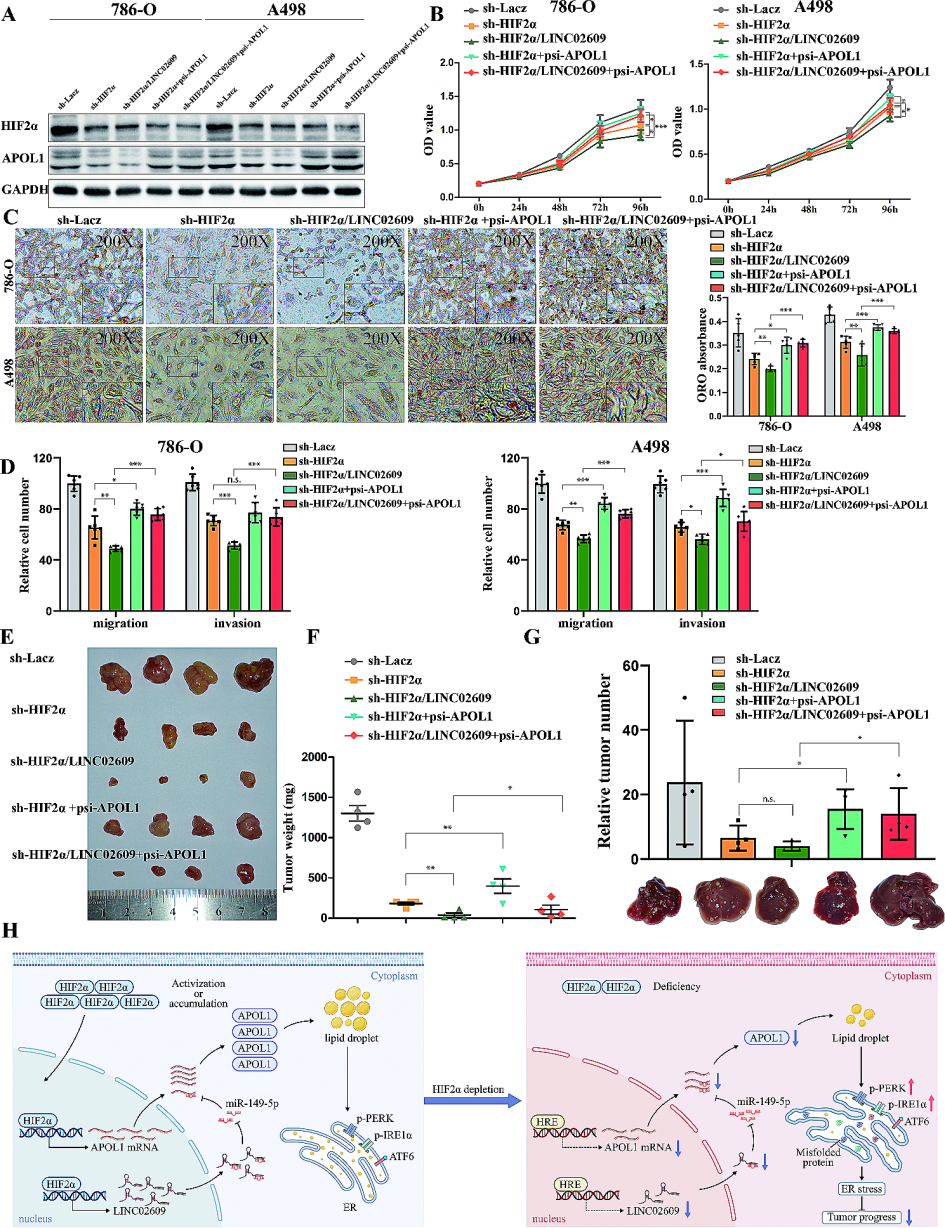
APOL1 partly reverses tumor progression and lipid storage initiated by HIF2α/ LINC02609 axis in vitro and in vivo. (**A**) WB analysis of HIF2α and APOL1 in renal cancer cell 786-O and A498 with indicated cells. (**B**) MTS assays revealed cell growth curves of indicated cells. (**C**) Photomicrographs of Oil Red O-stained in indicated cells (Magnification: 200× & 400×) and quantification of ORO in indicated cell lines. (**D**) Migration and invasion assays for indicated renal cancer cells. Number of migrated cells was quantified in three random images from each treatment group. (**E, F**) 786-O cells expressing indicated plasmid were transplanted into mice. Tumor weight of each nude mouse at the end of 49 days. (**G**) Representative images of livers of nude mice at the 30th days after IV. injection of indicated renal cancer cell. Quantification analysis of number of metastatic nodules. (**H**) Schematic model of HIF-2α/Apol1 mediated lipid storage promotes endoplasmic reticulum homeostasis and regulates tumor progression in ccRCC

To further determine whether APOL1 is a direct and functional mediator of the HIF2α /LINC02609 axis promoting tumor progression and lipid storage, we performed a rescue experiment by co-transfecting with HIF2α shRNA (versus the negative control), LINC02609 shRNA (versus the negative control) and APOL1 (versus the negative control) into 786-O and A498 cells. WB analysis showed that LINC02609 shRNA aggravated the suppression of APOL1 in HIF2α shRNA cell lines (Fig. 7A). In subsequent experiments, we found that LINC02609 shRNA aggravated the suppression of proliferation and metastasis by HIF2α and that APOL1 partly reversed the suppression of ccRCC by HIF2α shRNA or/and LINC02609 shRNA (Fig. 7B). We obtained similar results for lipid storage and metastasis mediated by HIF2α (Fig. 7C and D). We also transplanted the indicated cells into mice through subcutaneous transplantation and tail vein injection, similar to the proliferation and metastasis results in vivo. LINC02609 shRNA aggravated the suppression of proliferation and metastasis by HIF2α. More importantly, the expression of APOL1 reversed the proliferation and metastasis suppression phenotype induced by HIF2α knockdown (Fig. 7E-G) (Supplementary Fig. S13). We detected the expression of APOL1 in the subcutaneous transplantation and obtained the similar results (Supplementary Fig. S14). These results indicated that APOL1 can reverse tumor progression and lipid storage initiated by HIF2α/LINC02609 axis.

## Discussion

In the current work, we presented data demonstrating that APOL1 is involved in regulating renal cancer proliferation, metastasis and lipid storage both in vitro and in vivo. Of note, our data showed that lipid accumulation in ccRCC cells is VHL/HIF-2α dependent. In turn, our results indicated that HIF-2α can bind to the APOL1 promoter and regulate its expression directly. In addition, we also found that LINC02609, a HIF-2α-regulated long noncoding RNA could directly bind to miR-149-5p and effectively function as a sponge for miR-149-5p to modulate the expression of APOL1 in ccRCC (Fig. 7H). Further assays showed that APOL1 was upregulated and predicted poor prognosis in ccRCC. Depletion of APOL1 repressed tumor progression and lipid deposition in renal cancer. Additional bioinformatic analyzes and follow-up experiments indicated that APOL1-dependent lipid storage is required for ER homeostasis and cell viability in ccRCC. Thus, APOL1 is a critical target of the HIF-2α pathway involved in clear cell tumor progression and lipid storage.

There are important questions that need to be discussed.

How does the lipid droplet form? Cancer cells obtain lipids and lipoproteins through two mechanisms: uptake of exogenous lipids from their local microenvironment and *de novo* synthesis of endogenous lipid molecules. Lipid droplets (LDs), which are a prominent phenotype of ccRCC, are composed mainly of triglycerides (TGs) and cholesterol esters (CEs) [31, 43]. TGs consist of a glycerol backbone linked with three FAs, which can have various chain lengths and degrees of saturation [43]. Fatty acids (FAs) can be obtained from the diet or synthesized *de novo* [44]. In adult, normal, non-adipose tissues, the majority of FAs are acquired from the circulation, and *de novo* lipogenesis and expression of lipogenic enzymes are poorly expressed. In contrast, cancer cells exhibit a shift in lipid metabolism as most lipogenic enzymes are upregulated or activated [45]. Cancer-associated alterations in lipid metabolism include increased lipogenesis, increased lipid uptake from the extracellular microenvironment, and enhanced lipid storage and mobilization from intracellular lipid droplets (LD) [46].

CEs are the product of fatty acid esterification to cholesterol by acetyl-CoA acetyltransferase (ACAT). Cholesterol, a member of the sterol category of lipids, is a crucial component of cell membranes. In addition to being taken directly from the diet, cholesterol is synthesized through the mevalonate pathway. M. Celeste Simon et al. performed brilliant work and demonstrated that ccRCC cells suppress *de novo* cholesterol biosynthesis, despite accumulating high levels of cholesterol and cholesterol esters [47]. Therefore, cholesterol accumulation in ccRCC is more likely the result of increased uptake rather than excessive biosynthesis [48]. A master regulator of mevalonate pathway gene expression, sterol-regulatory element-binding protein 2 (SREBP-2) is key to maintaining cholesterol homeostasis [49]. How the large amount of lipid droplets accumulate in kidney cancer requires additional specific research.

What function does lipid droplets play? In solid tumors, hypoxia is a very general phenomenon. Hence, cancer cells show an expanded metabolic feature that affords the flexibility to withstand and grow in this harsh tumor microenvironment [50]. The first adaptive events in tumor metabolism to be identified are an exacerbated glucose uptake and glycolysis utilization leading to increased lactate production. Otto Warburg first observed an anomalous characteristic of cancer cell energy metabolism: even in the presence of oxygen, cancer cells can reprogram their glucose metabolism, and thus their energy production, by limiting their energy metabolism largely to glycolysis, leading to a state that has been termed ‘‘aerobic glycolysis’’[51, 52]. The existence of this metabolic switch in cancer cells has been substantiated in the ensuing decades. Such reprogramming of energy metabolism is seemingly counterintuitive, in that cancer cells must compensate for the 18-fold lower efficiency of ATP production afforded by glycolysis relative to mitochondrial oxidative phosphorylation [53]. This phenomenon suggests that tumors or tumor cells are not lacking in energy at all, and metabolic intermediates are more important for the development of tumors, or they have found some more efficient ways of generating energy?

Many human diseases, including metabolic, immune and central nervous system disorders, as well as cancer, are the consequence of alterations in lipid metabolic enzymes and their pathways [50]. Free FAs are critical for ATP production via β-oxidation. β-Oxidation is energetically very efficient (1 molecule of palmitate yields 129 molecules of ATP) but is O_2_ dependent and hence is extremely sensitive to blood flow [54]. ATP production via β-oxidation during hypoxia-reoxygenation was observed only in breast cancer cells. It seems that lipid droplets play a more important role in maintain internal environmental stability rather than energy supply in many tumors.

Four genes, CPT1A, PLIN2, CD36 and KLF6 have recently been implicated in HIF-dependent lipid accumulation in ccRCC [30, 55–57]. M. Celeste Simon et al. performed a preeminent study and found that PLIN2, a lipid droplet coat protein, is positively regulated by HIF2α, and promotes lipid droplet accumulation and ccRCC fitness [30].

Our RNA-seq data in OS-RC-2 cells(sh-Lacz vs. sh-HIFα) also hinted at this result (Supplementary Table S2). Unfortunately, the authors did not show how HIF2α regulates the expression of PLIN2, by direct binding to the promoter or other mechanisms. KLF6 driven by a robust super enhancer including the ccRCC-initiating VHL-HIF2α pathway supports the expression of the lipid metabolism regulators SREBF1 and SREBF2 [56]. These studies will help to integrate glycolysis and lipid metabolism with HIF2α in ccRCC. Cyril Corbet et al. found that TGFβ2-induced formation of lipid droplets supports acidosis-driven EMT and the metastatic spreading of cancer cells [58]. Yuan-Yuan Qu et al. found inactivation of the AMPK-GATA3-ECHS1 pathway induces fatty acid synthesis that promotes ccRCC growth [59]. Further work is thus needed for a comprehensive understanding of how different mediators of lipid metabolic phenotypes contribute to ccRCC.

Recent works indicate that cellular transformation commits tumors to growth programs that strain ER homeostasis, including dysregulation of protein and lipid metabolism [60–62]. Rapid tumor growth leads to hostile micro-environmental conditions, such as nutrient deprivation, oxygen limitation, high metabolic demand and oxidative stress, which disturb the protein folding capacity of the endoplasmic reticulum (ER), thereby provoking a cellular state of “ ER stress” [63]. ER stress triggers a dynamic signaling pathway known as the unfolded protein response (UPR). The UPR enforces adaptive or cell death programs by integrating information about the intensity and duration of stress stimuli [64]. One of our important findings is that APOL1 localizes to the ER in renal cancer. RNA-seq data and subsequent gene ontology (GO) enrichment analysis showed that the functions of APOL1 were most related to the intrinsic apoptotic signaling pathway in response to endoplasmic reticulum stress and the PERK-mediated unfolded protein response. The knockdown of APOL1 leads to ER expansion and activation of the ER sensors PERK and IRE1α. These results imply that APOL1 depletion leads to activation of the UPR and apoptotic cell death.

APOL1 is a hot research topic because it is strongly associated with nondiabetic CKD in black individuals [14, 17]. APOL1 has a membrane binding domain, and extracellular APOL1 is always bound to HDL particles; thus, it is logical that, when APOL1 is intracellular, it will also be associated with lipid-containing structures and organelles [65]. We and others have shown that APOL1 resides mainly in internal organelles, typically in the ER and mitochondria endosomes, lysosomes, and autophagosomes [18, 19, 66]. Justin Chun et al. also demonstrated that APOL1 localizes to intracellular lipid droplets (LDs). More importantly, this localization was not cell type specific, as was observed in Huh7, HeLa, and HEK-293 cells [20]. APOL1 association with LDs may be proportional to LD size or LD surface monolayer lipid composition. For this reason, the relationship between APOL1 and lipid metabolism is very close. In this article, we identified APOL1 as a key regulator of ccRCC progression. Patients whose tumors had high APOL1 expression had a shorter overall survival in ccRCC. HIF2α dependent APOL1 accelerates tumor growth in ccRCC cells through promoting lipid deposition and tumor progression. However, it remains to be elucidated how APOL1 affects lipolysis in ccRCC.

There are numerous studies on the function of APOL1 and its variants, as well as the downstream mechanisms of corresponding variants [14–16, 67–69]. However, little research has been conducted on the regulation of APOL1 expression in cancer. In this article, we found that HIF2α regulated the expression of APOL1 and LINC02609 directly by binding to its promoter. There were three lncRNAs, including LINC02609, LINC01320 and LICN01116, decreased simultaneously in both 786-O and A498 (sh-HIF-2α vs. sh-Lacz) by q-RT-PCR. Further study showed that knockdown LINC02609 or LINC01320 suppressed renal cancer cell proliferation and metastasis. What’s more, we found that knock-down LINC02609 expression can reduce the expression of APOL1 by WB and q-RT-PCR. LncRNA LINC02609 functions as a competing endogenous RNA to regulate APOL1 expression by sponging miR-149-5p in ccRCC. Above all, we found that APOL1-dependent lipid storage is required for ER homeostasis and cell viability in ccRCC.

How to reduce droplets content in ccRCC? Our previous study showed that lipid browning mediated by PLCL1/UCP1 promotes tumor cell “slimming” and causes abnormal lipid accumulation, which represses the progression of ccRCC [22]. We also found that the novel “tumor slimming” pathway mediated by melatonin/PGC1A/UCP1 exhibits prognostic potential in ccRCC [21]. By decreasing the amount of cell lipid droplets, tumor cell growth and metastasis can be significantly inhibited in ccRCC.

High lipid droplets (LDs) and stored-cholesteryl ester content in tumors are now considered hallmarks of cancer aggressiveness. LD-rich cancer cells are more resistant to chemotherapy [50]. Targeting the lipid and cholesterol dependence of cancer cell inhibitor agents directed against lipogenic enzymes (FASN, ACLY and ACC) has been the subject of numerous studies; and their efficacy as anticancer therapies has been proven in various preclinical models of carcinogenesis [70–72].

## Conclusions

In the current work, we presented data demonstrating that APOL1 is involved in regulating renal cancer proliferation, metastasis and lipid storage both in vitro and in vivo. Our studies identify HIF2α can regulate the expression of the lipid metabolism related genes APOL1 by direct and indirect means. Since ccRCC is considered a “metabolic disease” and “VHL hyper-mutation disease”, one unexplored avenue of controlling this tumor is to target the HIF2α/LINC02609/APOL1 pathway, which may offer a veritable therapeutic window.

### Electronic supplementary material

Below is the link to the electronic supplementary material.

Supplementary Figures

Supplementary Tables

## Data Availability

For all data requests, please contact the corresponding author (xzhang@hust.edu.cn) or the first author (xiaohaibing@ahmu.edu.cn).

## Abbreviations

ccRCC: clear cell Renal Cell Carcinoma
RCC: Renal Cell Carcinoma
TCGA: The Cancer Genome Atlas
LncRNA: Long Non-coding RNA
mRNA: Messenger RNA
KIRC: Kidney renal clear cell carcinoma
GEO: Gene Expression Omnibus
GO: Gene ontology
KEGG: Kyoto Encyclopedia of Genes and Genomes
GSEA: Gene set enrichment analysis
ChIP: Chromatin immunoprecipitation
FISH: Fluorescence in situ hybridization
VHL: von Hippel-Lindau tumor suppressor
HIF-2α: hypoxia inducible factor 2 subunit alpha
APOL1: Apolipoprotein L1
LINC02609: long intergenic non-protein coding RNA 2609
miR-149-5p: microRNA 149-5p
LD: lipid droplets
TGs: triglycerides
CEs: cholesterol esters
FAs: Fatty acids
ER: endoplasmic reticulum
ORO: Oil Red O
HRE: hypoxia response element
tTANRIC: The Atlas of Noncoding RNAs in Cancer
UPR: unfolded protein response
TEM: transmission electron microscopy
