# Supplementary material for: HIF-2α/LINC02609/APOL1-mediated lipid storage promotes endoplasmic reticulum homeostasis and regulates tumor progression in clear-cell renal cell carcinoma
Source: J Exp Clin Cancer Res. 2024 Jan 23;43:29. doi: 10.1186/s13046-023-02940-6 (PMC10804485; doi:10.1186/s13046-023-02940-6)
Supplement: Supplementary file 1 — Supplementary Figures [file 13046_2023_2940_MOESM1_ESM.docx]

**Supplementary Figures**


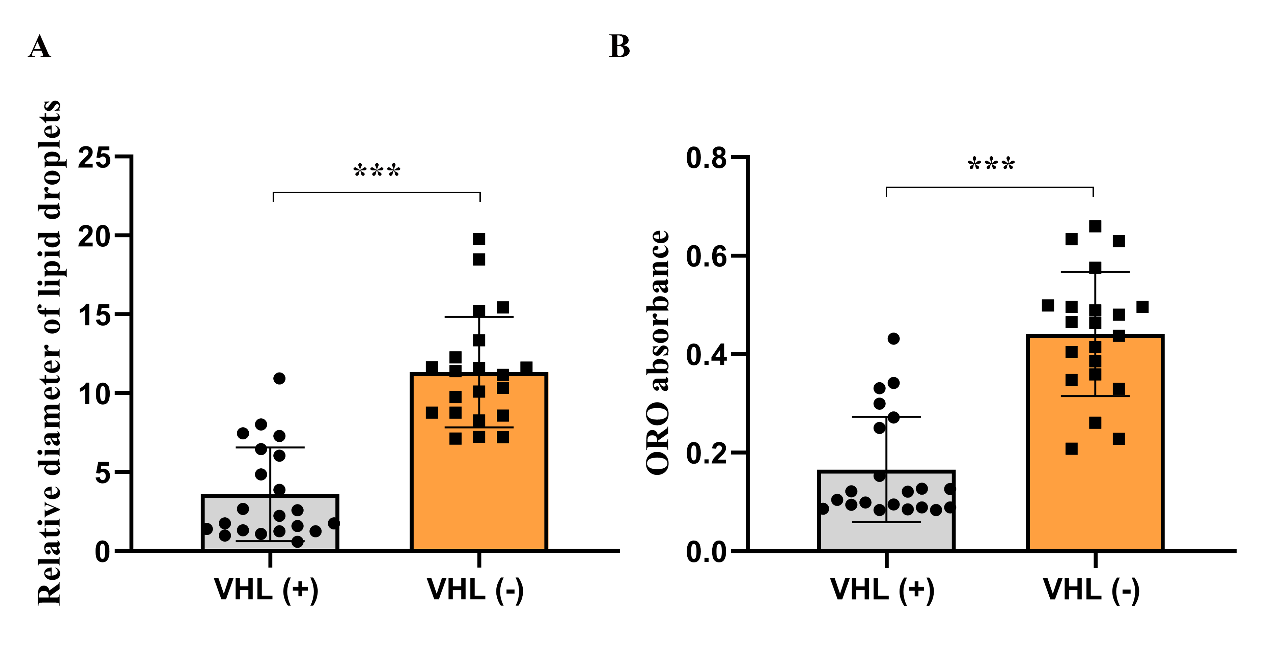


**Figure S1 The expression of lipid droplets in** **VHL (+) vs in VHL (-) ccRCC.** **(A)** Quantification of ORO in VHL(+) renal cancer cell lines and VHL(-) renal cancer cell lines. The data are presented as the means ± SEM. p values of two-tailed Student’s t tests are displayed. **(B)** Relative diameter of lipid droplets in VHL(+) renal cancer cell lines and VHL(-) renal cancer cell lines. The data are presented as the means ± SEM. p values of two-tailed Student’s t tests are displayed.


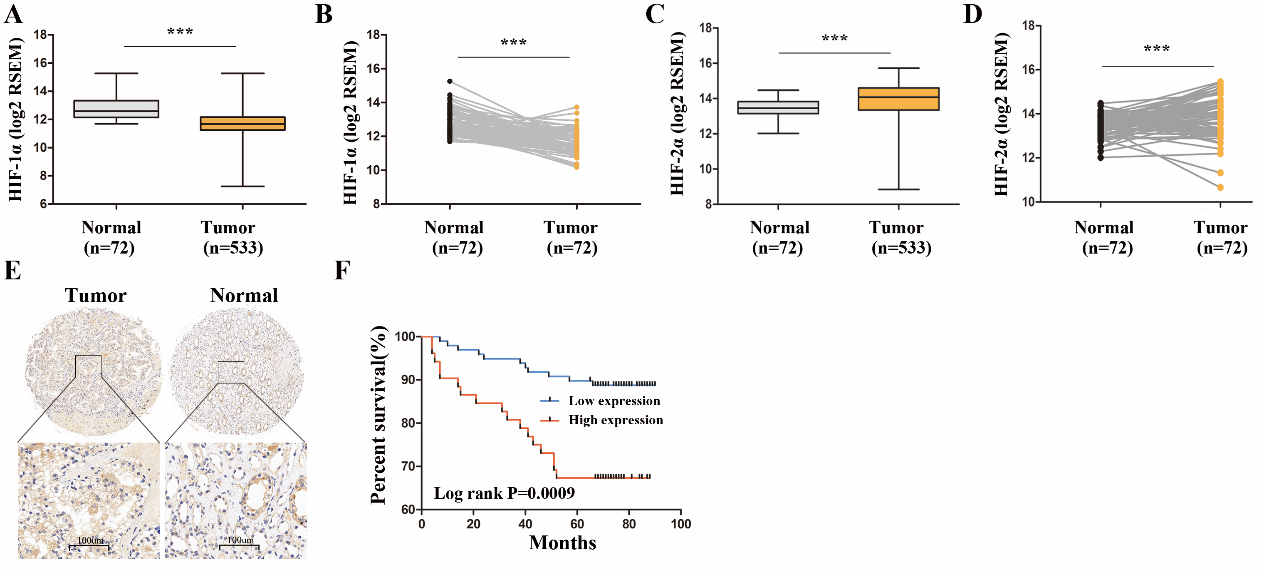


**Figure S2 The expression and prognosis of HIF1α and HIF2α in ccRCC. (A)** The expression of HIF1α in ccRCC (n=533) and adjacent normal kidney (n=72). The data were downloaded from the TCGA-KIRC dataset. **(B)** Relative expression of HIF1α in 72 pairs of ccRCC tumor tissues and their corresponding adjacent non-cancerous tissues. **(C)** The expression of HIF2α in ccRCC (n=533) and adjacent normal kidney (n=72). The data were downloaded from the TCGA-KIRC dataset. **(D)** Relative expression of HIF2α in 72 pairs of ccRCC tumor tissues and their corresponding adjacent non-cancerous tissues. The data were downloaded from the TCGA-KIRC dataset. **(E)** Representative images of HIF2α expression in ccRCC and adjacent normal kidney by IHC. **(F)** Kaplan-Meier curve showing overall survival of kidney cancer patients with high or low HIF2α expression (p=0.0009 by log-rank test).


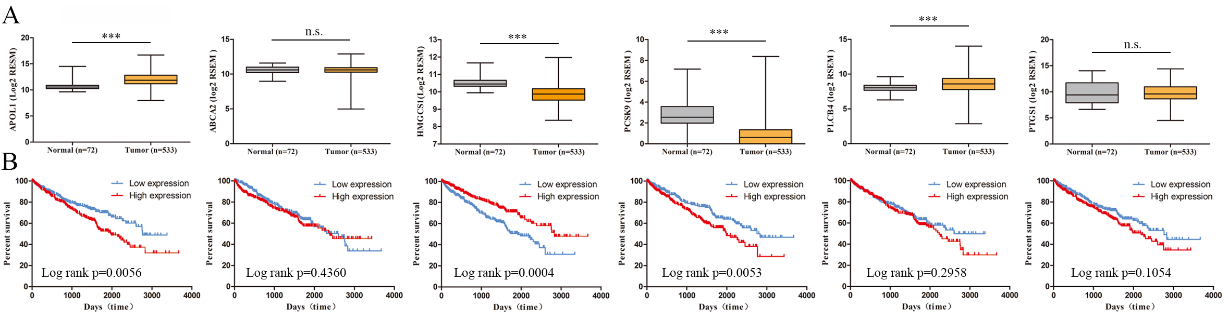


**Figure S3 The expression and prognostic analysis of HIF2α related molecules in TCGA-KIRC dataset. (A)** The expression of APOL1, ABCA2, HMGCS1, PCSK9, PLCB4 and PTGS1 in ccRCC (n=533) and adjacent normal kidney (n=72). The data were downloaded from the TCGA-KIRC dataset. **(B)** Kaplan-Meier curve showing overall survival of kidney cancer patients with high or low APOL1, ABCA2, HMGCS1, PCSK9, PLCB4 and PTGS1 expression.


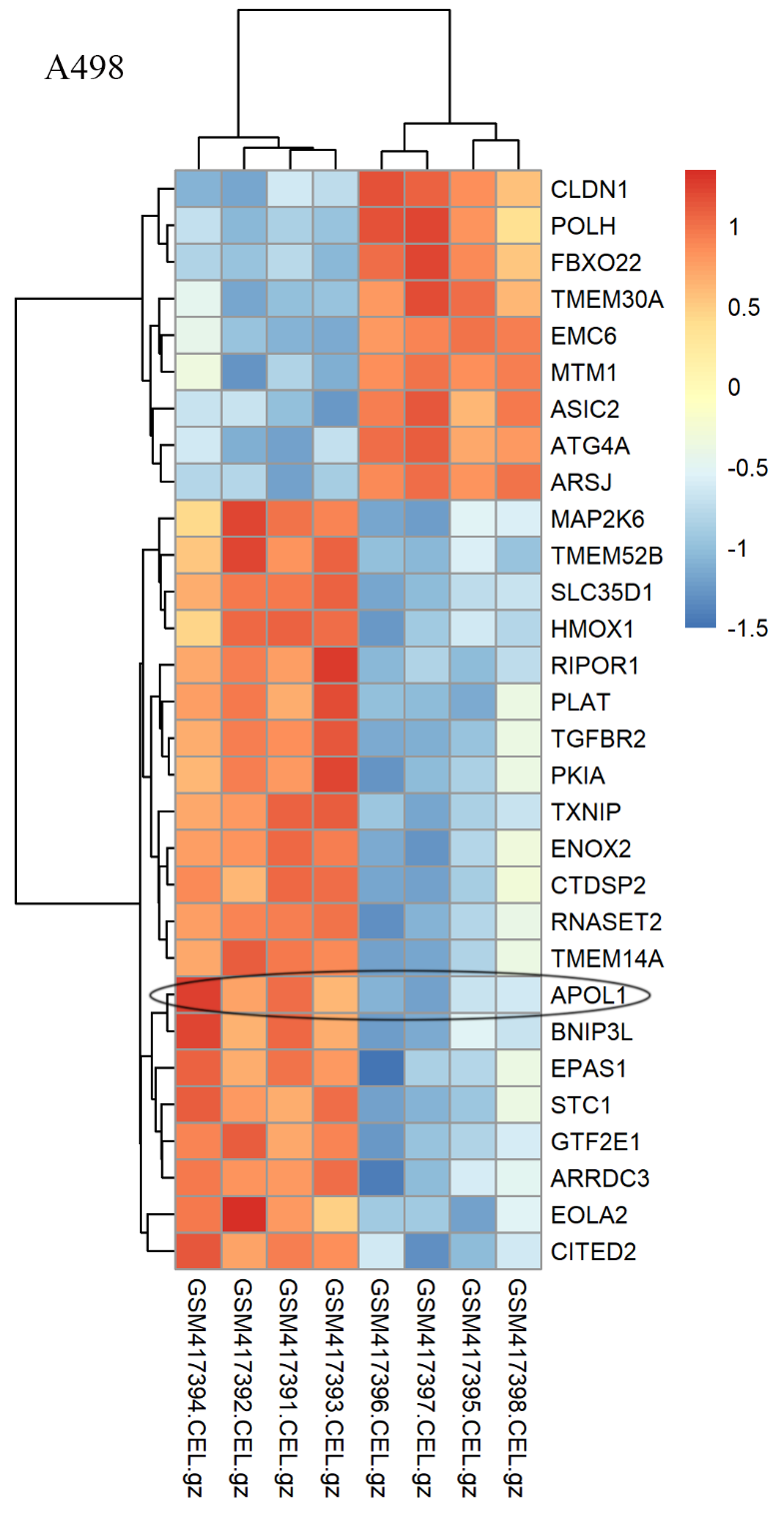


**Figure S4 The expression of HIF2α related molecules in A498 cell lines.** We retrieved and downloaded the GSE16622 dataset, which is a gene-expression datasets related to HIF-2α knockout in A498 VHL^(-/-)^ ccRCC cell lines from the GEO database. The "limma" package was utilized for differential analysis, and selected the top 30 genes with the most significant differences to draw heatmap.


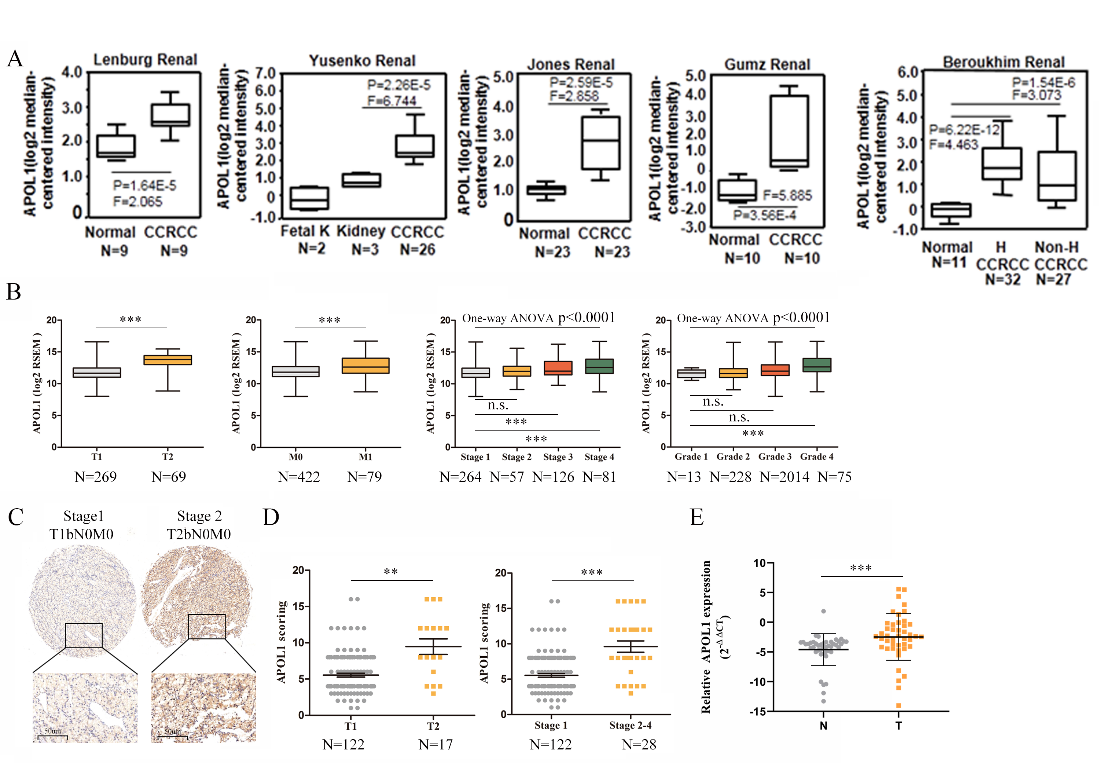


**Figure S5 The expression and clinical relevance of APOL1 in ccRCC. (A)** APOL1exist higher expression in tumor than in normal in renal cancer cell in Oncomine. **(B)** High APOL1 expression was significantly correlated with the tumor (T), node (N), grade and stage. One-way ANOVA were used to compare the difference among groups and two-tailed Student’s t tests to compare the difference between groups. **(C)** Representative images of APOL1 expression in Stage 1(T1bN0M0) and stage 2(T2bN0M0) in ccRCC by IHC. **(D)** High APOL1 protein expression was significantly correlated with the tumor (T), and stage. **(E)** The expression of APOL1 mRNA in cancer is higher than that in the adjacent tissues by q-RT-PCR. The tissues were form The First Affiliated Hospital of Anhui Medical University.


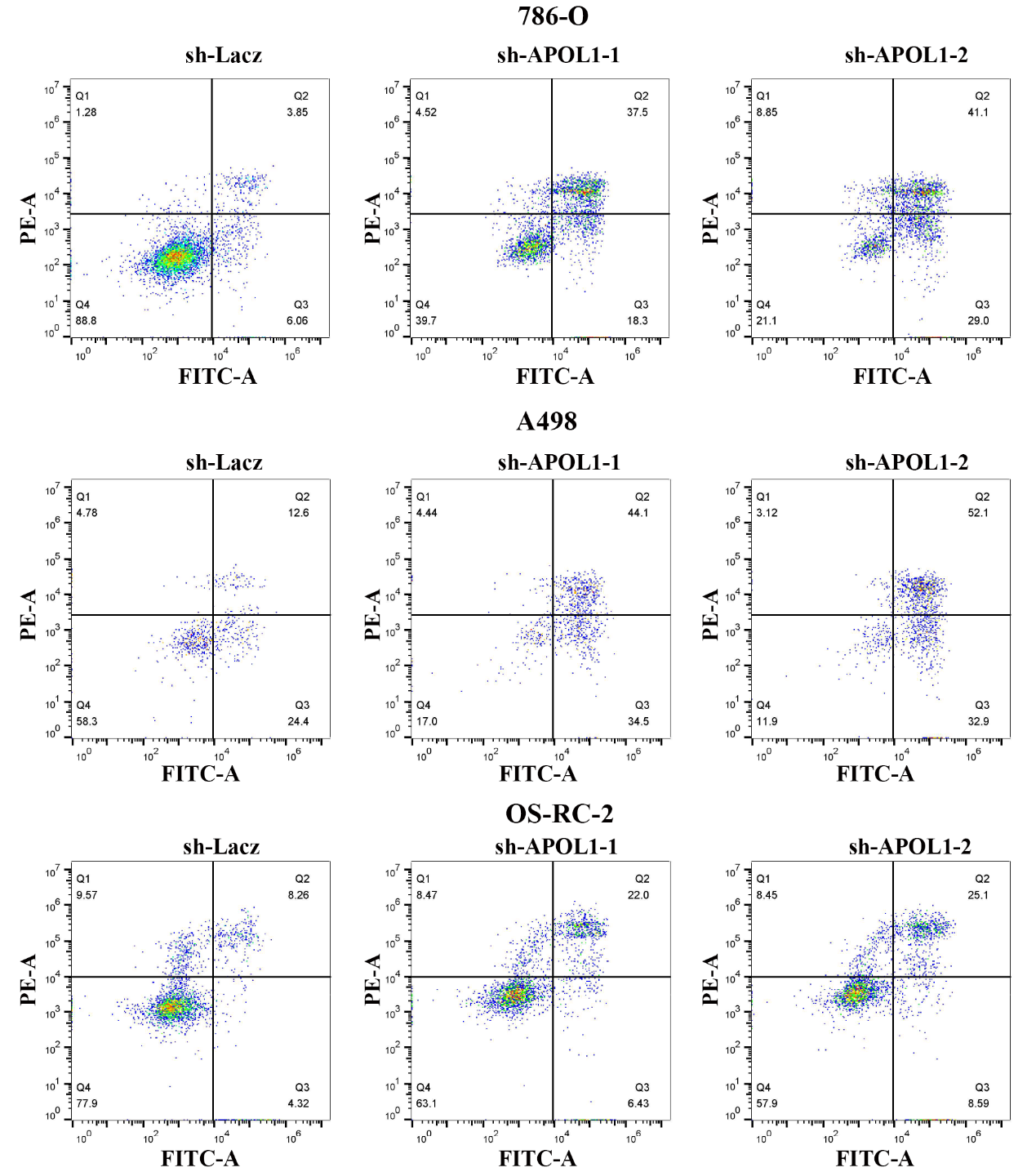


**Figure S6 Annexin V/PI double labeling assay revealed that APOL1 knockdown promoted cell apotosis in 786-O, A498 and OS-RC-2 cells.** Representative flow cytometry dot plots of 786-O, A498 and OS-RC-2 cells expressing indicated shRNAs. Early apoptotic cells (Annexin V-FITC positive, PI negative) were increased in sh-APOL1-1 and sh-APOL1-2 cells compared with control transfectants.


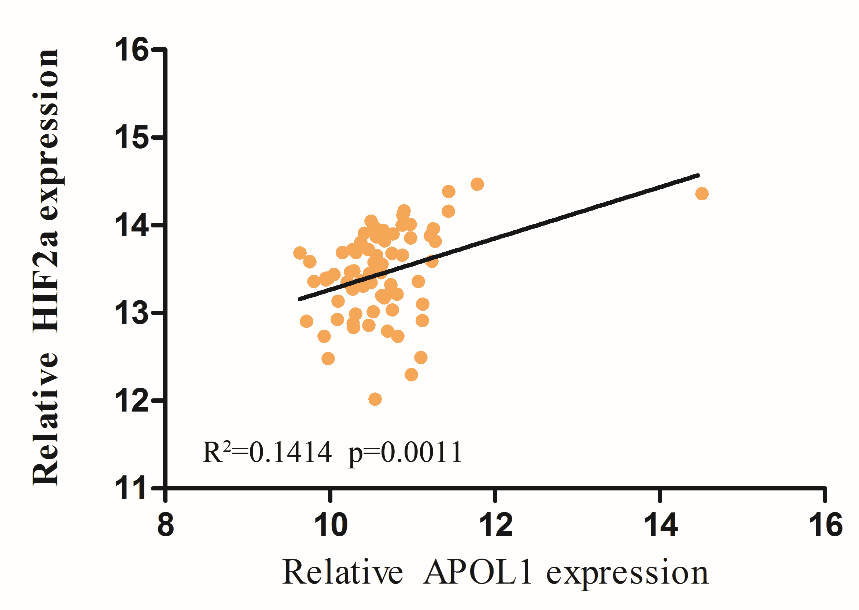


**Figure S7 The correlation analysis of the HIF2α and Apol1 genes in human normal renal tissue from TCGA Data Portal.** APOL1 expression correlates with HIF2α expression in human normal renal tissue from TCGA Data Portal (R^2^=0.1414, p=0.0011) (N=72).


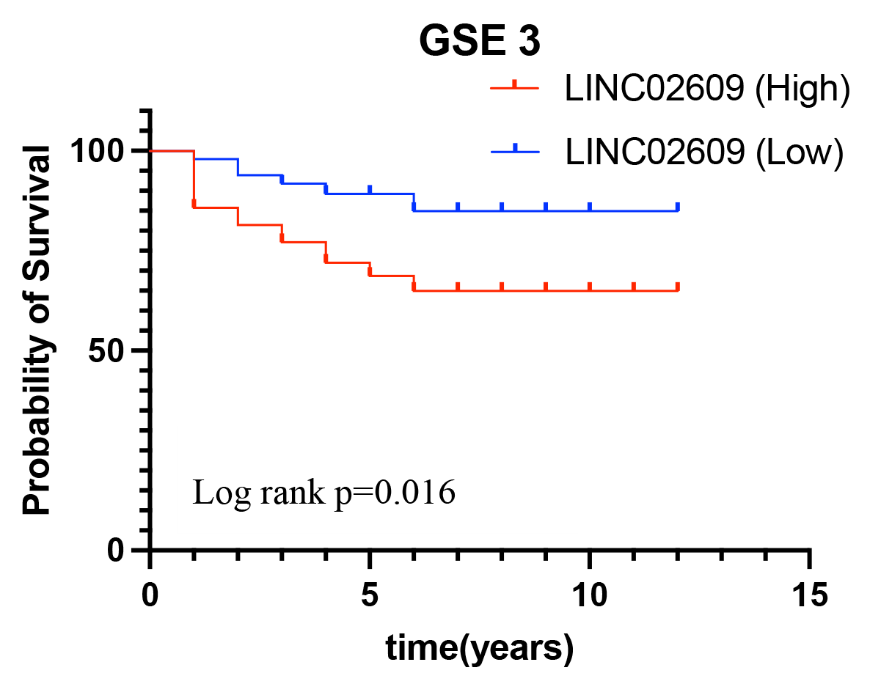


**Figure S8 LINC02609 -positive group showed significantly poorer overall survival than the LINC02609-negative group in GEO-GSE3.**


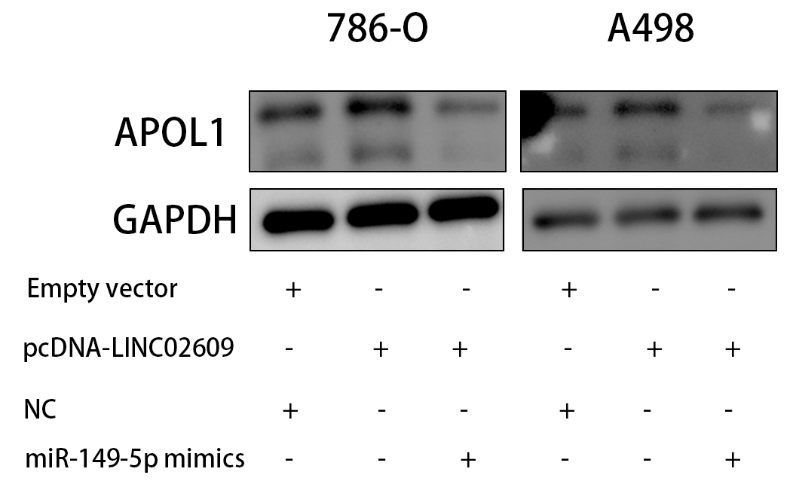


**Figure S9 LINC02609 overexpression trap hsa-miR-149-5p and rescue hsa-miR-149-5p-induced decrease of APOL1.** WB analysis of APOL1 in renal cancer cell 786-O and A498 with indicated cells transfected with pcDNA-LINC02609 or/and miR-149-5p mimics.


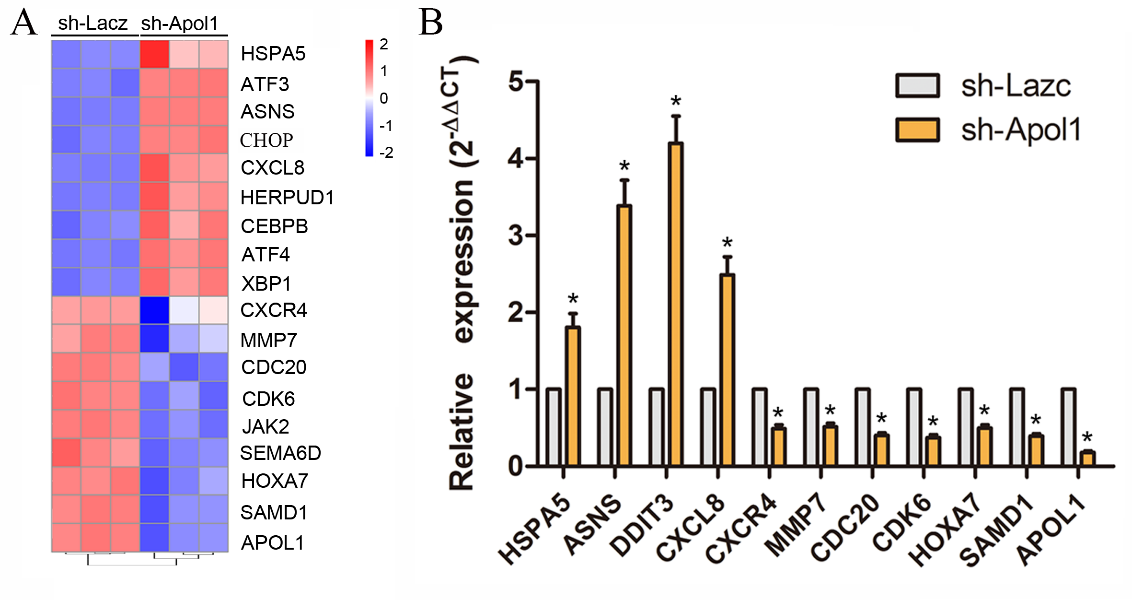


**Figure S10 The expression of Apol1 related molecules in 786-O. (A)** Heatmap showing the expression change of tumor progression related genes and UPR target genes in 786-O cells after transfection of APOL1 shRNA and control shRNA. **(B)** Q-RT-PCR analysis of tumor progression related mRNAs in renal cancer cell 786-O with APOL1 knockdown.


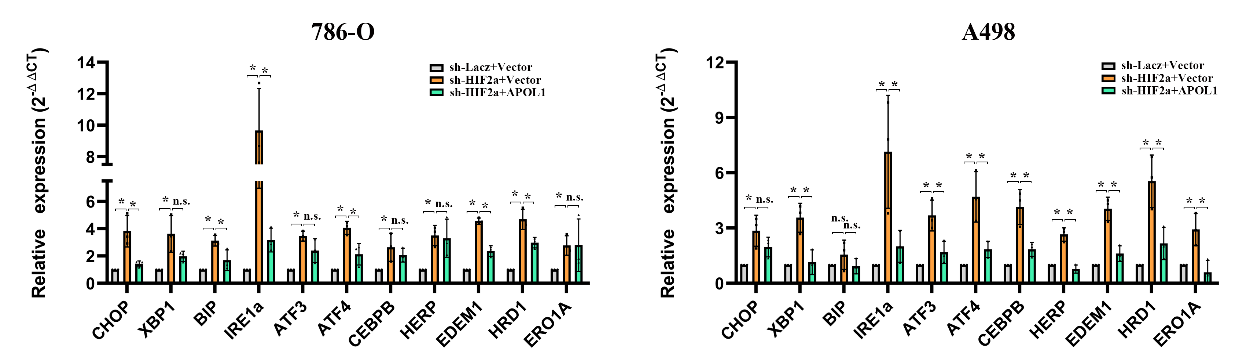


**Figure S11 The aggravated ER homeostasis caused by HIF2α can partly be reversed by APOL1 overexpression.** Q-RT-PCR analysis of UPR target genes in renal cancer cell 786-O and A498 with HIF2α knockdown and/or APOL1 overexpression.


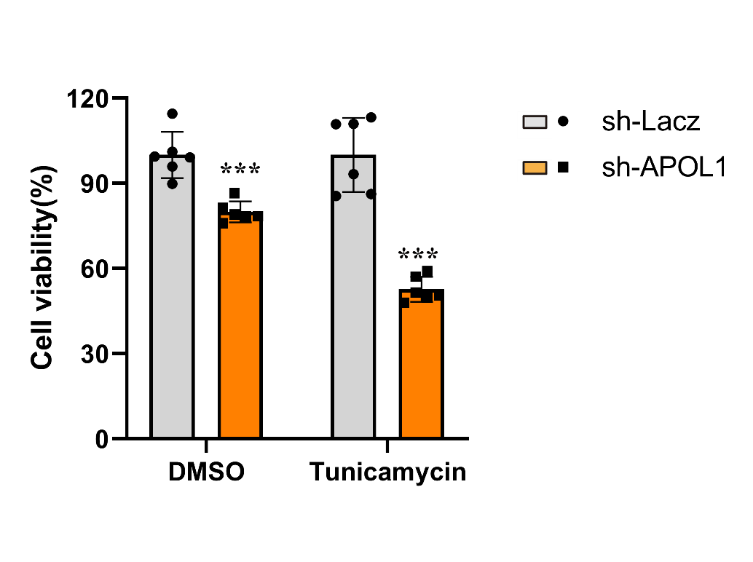


**Figure S12 MTS assay showed that APOL1-depleted cells were more sensitive to tunicamycin treatment, compared with controls in renal cancer cell lines 786-O.**


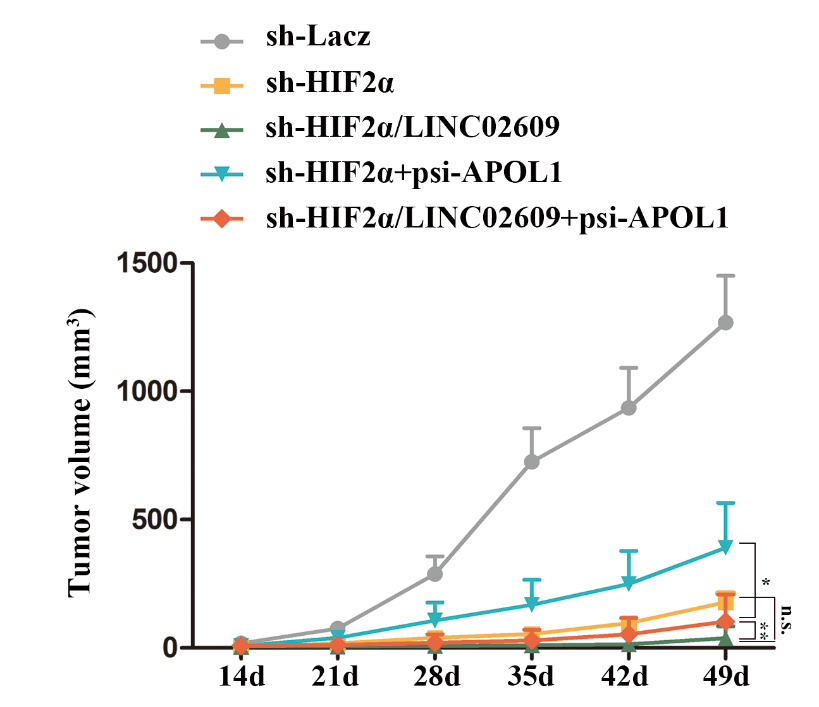


**Figure S13 APOL1 partly reverse tumor progression initiated by HIF2α/ LINC02609 axis *in vivo*** Mean tumor volume measured by caliper on the indicated days.


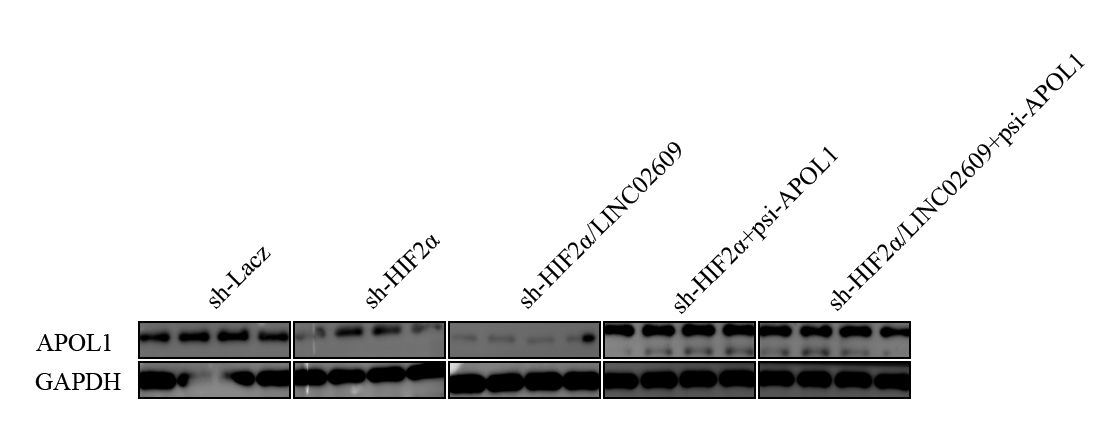


**Figure S14 The APOL1 expression in xenograft tumor samples.** WB analysis showed that LINC02609 shRNA aggravated the suppression of APOL1 in HIF2α knock-down xenograft tumor samples.
